# Enhancing Triboelectric Nanogenerator Performance via Ultraviolet Nanosecond Laser-Engineered Microstructured Intermediate Layer

**DOI:** 10.1021/acsami.5c13450

**Published:** 2025-09-18

**Authors:** Zheng Zheng, Lei Li, Mingming Liu, Liyong Wang, Ruiyong Yang, Rui Li, Langping Wang, Hongyu Zheng, Youbin Zheng

**Affiliations:** † School of Mechanical Engineering, 91620 Shandong University of Technology, Zibo 255000, China; ‡ Department of Electrical Engineering and Electronics, 4591 University of Liverpool, Liverpool L69 7GJ, U.K.; § State Key Laboratory of Precision Welding & Joining of Materials and Structures, 47822 Harbin Institute of Technology, Harbin 150001, China

**Keywords:** triboelectric nanogenerator, ultraviolet nanosecond laser, intermediate layer, enhanced performance, energy harvesting

## Abstract

The increasing demand for powering wireless sensors and wearable electronics has intensified the need for efficient and sustainable powering solutions. In this context, triboelectric nanogenerators (TENGs) have emerged as a promising technology for sustainable power supply, converting ambient mechanical energy into electricity. To improve energy harvesting efficiency and durability, it is crucial to continuously enhance output performance and extend cycle life. Introducing an intermediate layer has proven effective in boosting TENG performance, however, most existing designs remain planar structures and do not fully maximize the charge storage potential of the intermediate layer. In this work, we introduced a novel approach to improving TENG performance through ultraviolet nanosecond laser-engineered intermediate layers. This method significantly enhanced TENG output, increasing the short-circuit current by 3.8 times (from 2.2 to 8.4 μA) and the open-circuit voltage by 2.3 times (from 160 to 370 V) compared to TENGs with flat intermediate layers. The improvement is attributed to an increased effective bonding area, which enhances charge storage capacity. In addition, the generated graphite oxide layer during processing and the laser heat affected layer on the underside of the polyimide intermediate layer accelerates charge accumulation and the charging speed increased by 3 times. We also systematically investigated the influence of laser power, speed, processing patterns, and intermediate layer thickness on TENG performance. The results show that the appropriate matching of laser parameters and the dielectric layer thickness can maximize TENG performance.

## Graphical abstract

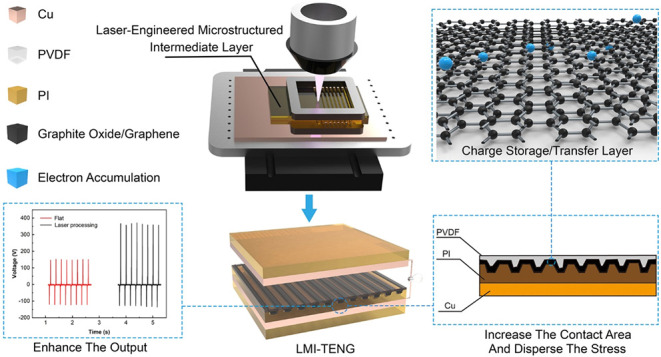

## 1 Introduction

With the development of science and technology, human society has gradually entered a new era of big data and the Internet of Things (IoT). Research on flexible electronic devices, sensor networking, and blue energy collection have become the hotspots.^1–4^ Alongside these research, energy consumption by these millions of portable/wearable devices has become an urgent issue. Portable energy harvester that can collect renewable mechanical energy from the environment, or to use it in a lightweight form to replace batteries, providing a stable and continuous supply of electrical power at the microwatt (μW) to milli-watt (mW) level for these electronic devices, are highly desirable. Triboelectric nanogenerator (TENG) is such an effective energy harvester, which converts the ambient mechanical energy into electrical energy based on triboelectrification and electrostatic induction effects. To enhance energy harvesting efficiency and enable them to power a broader range of electronic devices, continuous improvements in output performance,^5,6^ cycle life,^7^ and applicability^8,9^ are essential.

A lot of efforts have been made to enhance the output performance of TENGs, mainly focusing on increasing charge generation through nano/microstructuring of the triboelectric layer, triboelectric material selection with greater disparity in electron donation and acceptance capabilities, and chemical modification. While these strategies can improve the output performance, the surface structures and chemical modification of the triboelectric layer are prone to wear during the friction process, especially the nanostructured and modified layers. To address this limitation, Cui^10^ introduced a novel intermediate layer between the triboelectric layer and the electrode to store charges generated during the triboelectrification process and prevent charge recombination, thereby enhancing the output voltage and current. Subsequent studies explored various materials for the intermediate layer, including polyimide (PI),^11^ ion-containing electrolyte polymers,^12^ MoSe_2_ nanosheets,^13^ MXene/silicone.^14^ In addition, Xin et al.^15^ studied the influence of single and double-sided intermediate layers on output, pointing out that the coupling effect of positive and negative intermediate layers would reduce the output. Therefore, most current studies choose a single-sided intermediate layer. In a subsequent study, Song et al.^16^ have systematically studied the influence of the thickness of the dielectric layer on the output of TENG, demonstrating the existence of the optimal thickness of the TENG dielectric layer. These findings also provide ideas for this paper, that is, to consider not only the solely thickness of the triboelectric layer and the intermediate layer, but also the total thickness of the dielectric layer including the triboelectric layer and the intermediate layer in order to better enhance the output of TENG. These studies highlight that the primary role of the intermediate layer is to enhance charge trapping and reduce charge decay. Although different intermediate layer materials and composite intermediate layers have been reported for improving the output of TENG, most designs remain planar structures and do not fully maximize the charge storage potential of the intermediate layer. Few studies have been conducted to explore the impact of microstructured intermediate layers on TENG performance. A systematic study on microstructured intermediate layers could unlock new opportunities for optimizing TENG efficiency, which eventually leading to a new avenue for boosting TENGs performance.

In recent years, laser technology has emerged as an advanced processing tool in the additive processing,^17^ removal processing,^18^ and surface modification^19–21^ of materials, etc. In the field of TENG fabrication, laser techniques have been used to pattern triboelectric layers, increasing contact area and enhancing energy output.^22–26^ However, most research has focused on structuring the triboelectric layer or electrodes, with limited attention given to laser processing of intermediate layers. Salauddin et al.^27^ applied laser carbonization to MXene/ZiF-67 nanocomposite intermediate layers, optimizing laser power and speed parameters to improve charge retention. Moreover, research on enhancing TENG output through laser processing has mostly centered on femtosecond and continuous lasers, with little exploration of nanosecond laser processing of intermediate layers. However, the industrial application of nanosecond lasers is more mature. On the one hand, they offer advantages such as higher processing efficiency and lower costs compared to femtosecond and picosecond lasers. On the other hand, the short wavelength of ultraviolet laser and the short nanosecond pulse are conducive to the material absorbing laser energy and reducing the influence of thermal effects, which can avoid the serious thermal effect problems generated during the processing of continuous laser or long pulse laser,^28–30^ making them suitable for large-scale application.

In this paper, we employed ultraviolet nanosecond laser processing to fabricate microstructures on the intermediate layer and investigated their influence on modulating the output performance of the TENG. The short-circuit current of the TENG with laser-engineered microstructured intermediate layer (LMI-TENG) was increased by 3.8 times from 2.2 to 8.4 μA, and the open-circuit voltage was increased by 2.3 times from 160 to 370 V, comparing to the TENG with flat intermediate layer (FI-TENG). Benefiting from this nanosecond laser processing approach, the effective bonding area between the intermediate layer and the triboelectric layer was increased, enhancing the charge storage capacity and output performance. In addition, we systematically investigated the effects of laser power, speed, and processing patterns on TENG output and analyzed the impact of processing different thicknesses of the PI intermediate layer on TENG performance. This work provides a new angle into configuring intermediate layers to enhance TENG performance, offering a new approach to improving both the cycle life and output performance of TENGs.

## 2 Experimental Section

### 2.1 Materials

PVDF (Arkema, *M*_w_ ∼ 500,000, powder), PI films (with thicknesses of 15, 25, 50, 75, and 100 μm), graphene oxide solution (oxidation degree 36%, flake diameter 10 μm), copper tapes, resistors and capacitors are purchased from the domestic market. All other chemicals are analytical grade reagents and can be used without further treatment.

### 2.2 Experimental Setup

The nanosecond ultraviolet laser (Coherent, USA) has a wavelength of 355 nm, a pulse width of 10 ns, and a repetition frequency of 200 kHz. Figure 1a shows a schematic diagram of the experimental setup used for nanosecond laser processing. The laser first passes through a half-wave plate, then through a beam splitter, which separates the required resonant light. It is then expanded and collimated by a beam expander, reducing the divergence angle. The beam is further directed through a shutter, a galvanometer scanner, and an objective lens (50×, NA 0.16) before finally focusing on the sample surface for processing. The laser intensity irradiated on the sample surface can be controlled by adjusting the half-wave plate. The output time of the laser is controlled by the shutter. The movement of the laser focus is realized by the oscillation of the galvanometer. Additionally, a translation stage with a maximum speed of 2 mm/s is used, which can adjust the *Z*-axis before processing to achieve positive and negative defocusing of the laser. During processing, the worktable remains stationary while the beam moves to process the material.

**Figure 1:**
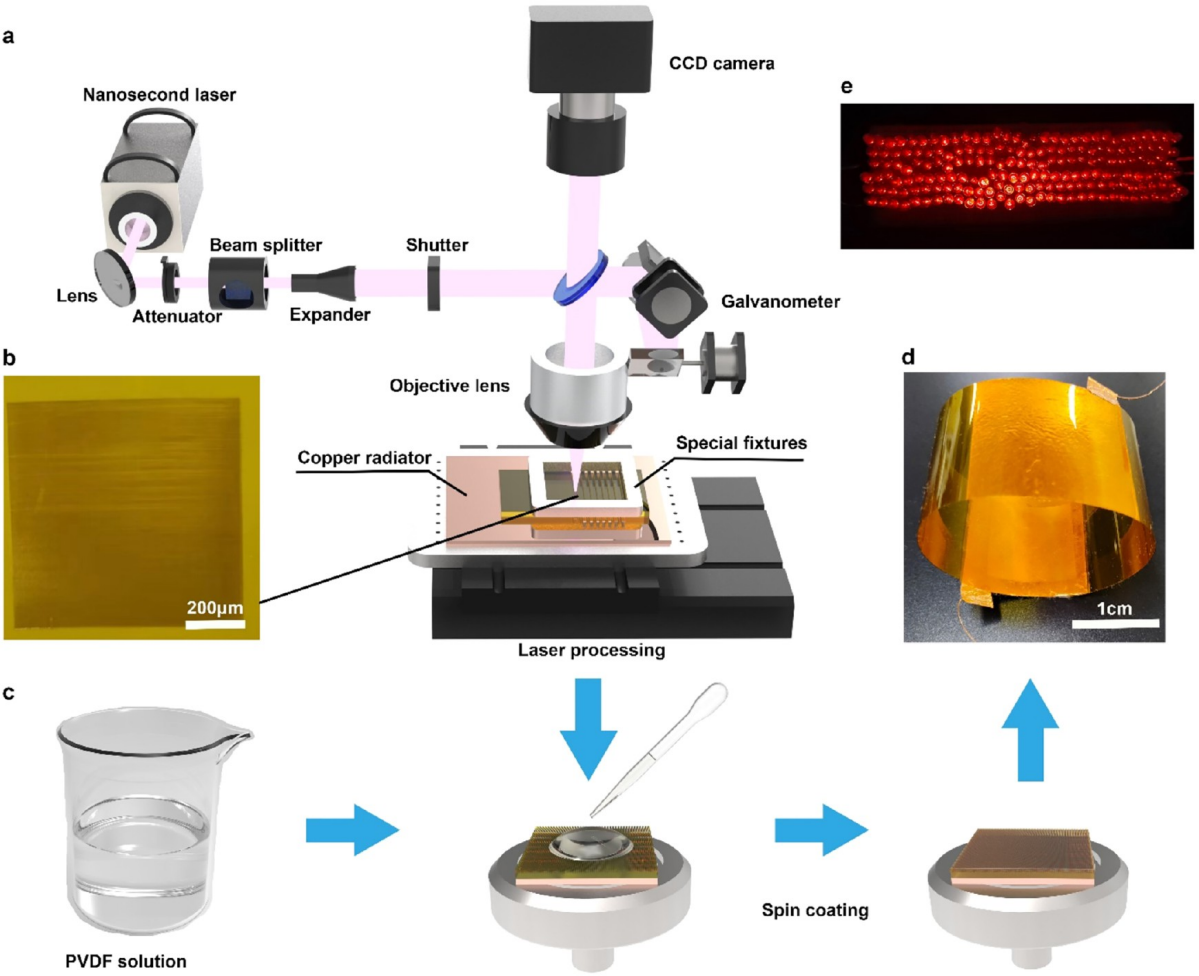
(a) Schematic diagram of laser optical path and processing. (b) The image of PI film after ultraviolet nanosecond laser processing. (c) Schematic illustration of fabrication process. (d) The photo of fabricated TENG. (e) The LEDs powered by TENG.

### 2.3 Fabrication of Microstructures

To fabricate microstructures on the intermediate layer, the PI film and the underlying heat-dissipating copper sheet are secured onto a three-axis motorized stage using a specialized fixture. The laser is then adjusted to the focal position, with a focal spot diameter of 5 μm. A preprogrammed process is executed to fabricate linear and bowl-shaped microstructures on the PI surface, as shown in Figure 1b. Two methods are used to fabricate bowl-shaped structures. The first employs a single-pulse ablation technique, where the laser scanning speed and frequency are adjusted to ensure that the interval between pulses exceeds the spot diameter. This method is suitable for processing bowl-shaped structures smaller than 10 μm. The second method is multipulse ablation, which entails designing a circular array pattern and filling the circular pattern with laser pulses. This ablation method enables the processing of bowl-shaped structures ranging from 10 to 100 μm. Under different power densities, the PI film may undergo melting, sintering, or graphene oxide formation due to photothermal conversion. In order to obtain microstructures of different sizes and morphologies, it is necessary to control the laser scanning speed within 50 to 1500 mm/s and the laser power within 0.10 to 4.20 W.

### 2.4 Characterization

The surface patterns and qualities after laser processing are characterized by an ultradepth field microscope (OLYMPUS, DSX1000), and the thickness of the TENG structure and the surface morphology of the intermediate layer are characterized by scanning electron microscope (SEM, quantum 250 FEG, Thermo Fisher Scientific). An energy spectrometer (EDS) is used to examine the element change in the cross-section after laser processing. Then, a Raman spectrum (Raman shift 1000–3000) is recorded on a Renishaw Raman microscope using a 532 nm laser with a power of 5 mW. X-ray photoelectron spectroscopy (XPS) is performed using a Thermo fisher Nexsa SXM scanning X-ray microprobe with a base pressure lower than 5 × 10^–8^ Torr. Raman and XPS are used to analyze the changes in surface composition, defects, and functional groups. Atomic force microscopy (AFM) (Bruker Dimension ICON) is used to evaluate the Young’s modulus of the laser-engineered intermediate layer. For TENG performance characterization, a commercial linear mechanical motor is used to drive the fabricated TENG at a contact frequency of 5 Hz and a stroke of 25 mm (with an actual contact area of 15 mm × 15 mm). The linear motor applies an external force to the TENG, generating friction between two triboelectric layers, thereby producing triboelectric potential and electrical output in the external circuit. The impact force generated by the linear motor is controlled through the acceleration of a thrust rod and measured by a digital push–pull force gauge (SUNDOO, SH 500 N). The open-circuit voltage of the TENG is measured using an oscilloscope, while the short-circuit current is recorded with a Keithley 2450 precision digital source meter. For strain testing, resistive strain gauges (produced by Chengdu Electronic measurement and sensing technology) are used to measure the strain of the PVDF layer under different pressures.

### 2.5 Assembly of TENG

PVDF powder is dissolved in *N*,*N*-dimethylformamide as the solvent to prepare a 10 wt % PVDF solution at 60 °C under magnetic stirring (Figure 1c). A copper foil is used as the tribo-positive layer, as well as the electrode (15 mm × 15 mm). On the nonstructured side of the laser-processed PI, another piece of copper foil is attached, serving as the electrode of the tribo-negative layer. The prepared PVDF solution is spin-coated on the structured PI surface serving as the tribo-negative layer, then vacuum-dried in an oven at 80 °C for more than 2 h to remove the residual solvent. The thickness of the triboelectric layer is controlled by adjusting the spin-coating speed (900–2100 rpm). The TENG automatically recovers its shape after being pressed on one side with a copper-coated wire as a lead (Figure 1d).

## 3 Results and Discussion

To investigate the role of microstructured intermediate layer in regulating the TENG output performance, an ultraviolet nanosecond laser processing technique (Figure 1) was employed to fabricate the intermediate layer with microstructures. By exploring laser processing parameters and spin-coating conditions, the influence of laser processed PI intermediate layer on TENG performance was analyzed, ultimately enabling the fabrication of TENGs with high-performance output. As shown in Figure 1e, the optimized TENG successfully powers 200 commercial LEDs.

### 3.1 The Working Principle of LMI-TENG

Cu and PVDF were selected as the triboelectric materials to fabricate TENGs. Since PVDF is positioned at the negative end of the triboelectric series, it readily gains electrons when in contact with a relatively positive materials like Cu. In initial state (Figure 2a­(I)), the PVDF and upper Cu layer are separated. When pressed into contact, positive charges are generated on the Cu surface due to the triboelectric effect, while negative charges appear on the PVDF surface (Figure 2a­(II)). The negative charges on PVDF, apart from some that drift and neutralize, are retained on the PVDF surface or are captured and stored by electron traps within the laser-processed intermediate layer. Upon separation, electrostatic induction between the PVDF–PI intermediate layer and the lower Cu, along with the potential difference between the two Cu layers, drives a current from the upper Cu through an external load to the lower Cu electrode (Figure 2a­(III)). Once the charges reach an equilibrium, there is no current flow in the external circuit (Figure 2a­(IV)). Similarly, when the upper Cu and PVDF come into contact again, the current flows from the lower Cu electrode through the external load back to the upper Cu tribo-layer to balance the charge between the upper Cu and the PVDF-intermediate layer (Figure 2a­(V)). Finally, this continuous contact-separation process results in alternating electricity generation.

**Figure 2:**
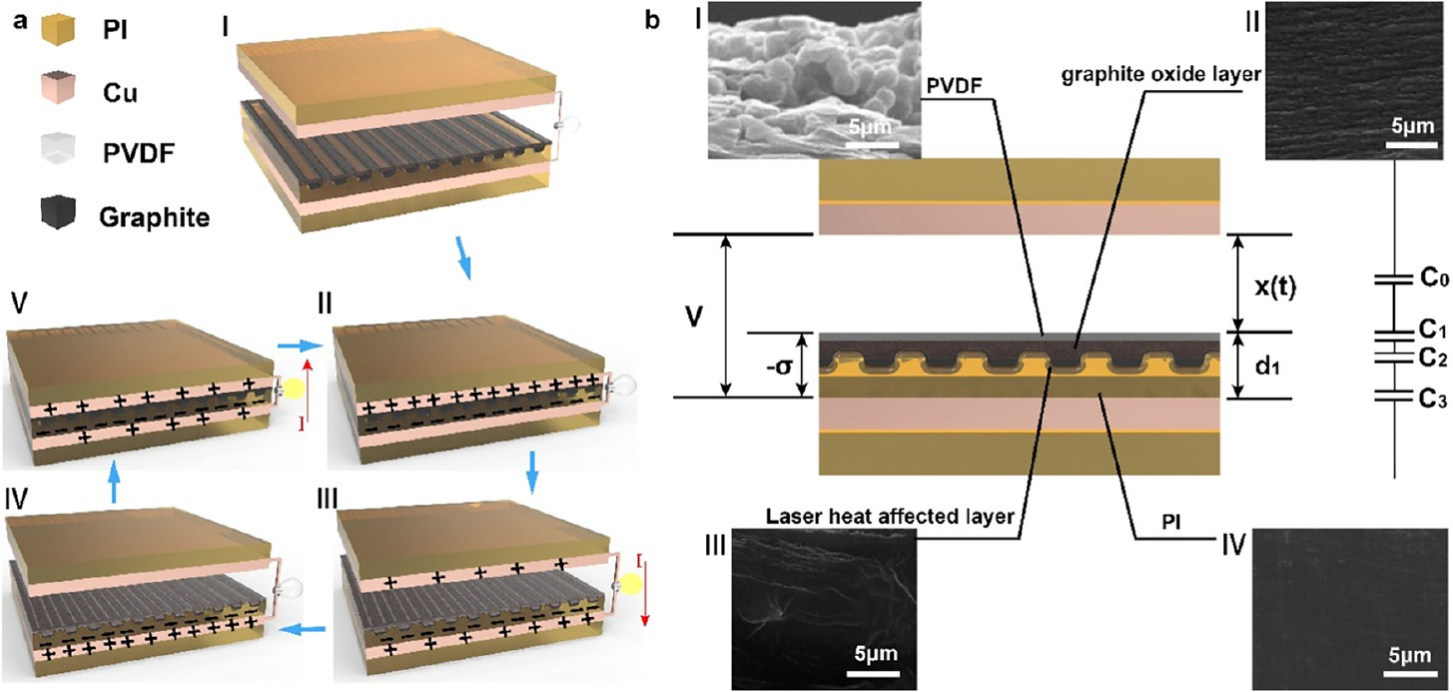
(a) Schematic diagram of the working mechanism and charge generation of LMI-TENG, (b) the structural diagram and equivalent circuit model of LMI-TENG with a laser-processed microstructured PI film as the intermediate layer.

In the aforementioned process, the main factor affecting TENG output is the amount of charge transfer, which is determined by charge generated and charge decay. Charge generation is primarily influenced by the tribo-layers, and when the amount of charge generated is constant, enhancing the charge retention is also a strategy to reduce charge decay and improve output.^10^ By adding an intermediate layer, a portion of the triboelectric charge generated in the PVDF can be stored, thus reducing charge drift and neutralization within the PVDF. This increases the amount of charge retained on the surfaces of the PVDF and the intermediate layer.^11^ Consequently, in subsequent contact processes, this leads to a greater potential difference between the two Cu electrodes, inducing more charge during separation and resulting in a higher external current. However, there is a limit to the amount of charge that PVDF and the intermediate layer can store; with continuous contact and separation, charge storage will reach saturation, limiting further enhancement of TENG output. Laser processing can create microstructures and additional electron traps on the intermediate layer, allowing greater charge accumulation and storage. As shown in Figure 2b, the LMI-TENG, compared to the unprocessed original FI-TENG, has an additional oxidized graphite layer (Figure 2b­(II)), also referred to as the PVDF-oxidized graphite hybrid layer, along with a laser-induced thermal effect layer beneath it (Figure 2b­(III)). Here, the TENG system can be modeled as a series of capacitors, where the dielectric materials serve as the dielectric medium. Figure 2b illustrates the equivalent circuit model of the LMI-TENG. Referring to Gauss’s law, its voltage output can be expressed by the following eq 1.^31^

1 $$V=E_{1}d_{1}+E_{\mathrm{air}}x=-\frac{Q}{S\varepsilon_{0}}\left(\frac{d_{1}}{\varepsilon_{1}}+x\left(t\right)\right)+\frac{\sigma x\left(t\right)}{\varepsilon_{0}}$$

Where *d*_1_ is the thickness of the triboelectric layer and the intermediate layer, and ε_1_ is their relative dielectric constant. The amount of charge transferred between the two electrodes driven by the induced potential is *Q*. The distance *x*(*t*) between the PVDF and Cu triboelectric layers changes with the contact-separation due to the mechanical force. After the contact, the Cu triboelectric layer and the PVDF surface will have opposite static charge densities σ. Due to the change in the surface properties of the intermediate layer dielectric material caused by laser processing, the capacitance of the equivalent capacitor will change accordingly. As we all know, the charge *Q* is proportional to the product of the output voltage *U* and the capacitance *C*, as shown in eq 2.

2 Q=U·C

Here, the thickness of the dielectric layer and the distance between the triboelectric layers are not on the same scale, and the change in distance *x*(*t*) determines the overall separation. Since ε_0_ defines the dielectric constant, the variation in capacitance can be considered negligible. Experimental results demonstrate that nanosecond laser processing of the PI intermediate layer significantly enhances the open-circuit voltage and short-circuit current of the TENG. Figure 3 represents the performance of LMI-TENG under optimal parameter conditions. As shown in Figure 3a, after multiple contact-separation cycles until saturation, the measured short-circuit current of LMI-TENG significantly increases, indicating the amount of induced charge increased. The FI-TENG produced a maximum current of 2.2 μA after 30 min of accumulation, while LMI-TENG achieved a maximum current of 10.2 μA after 45 min of accumulation. This indicates that LMI-TENG not only stores more charge but also exhibits a significantly faster initial charge accumulation rate compared to FI-TENG. Figure 3b,3c compare the output performance of the two TENGs after an accumulation period of 30 min. The short-circuit current of LMI-TENG increased from 2.2 to 8.4 μA, and the open-circuit voltage increased from 160 to 370 V. As shown in Figure S1, compared with the FI-TENG, the surface charge density of the LMI-TENG increases more significantly with the increase in frequency. This indirectly indicates that the laser-processed PI intermediate layer improves the charge storage/transfer efficiency, enabling the entire dielectric layer to accumulate charge faster and in larger quantities. Wu et al.’s research indicates that it is difficult for the triboelectric materials of TENGs to reach their maximum storable charge density in tests.^32^ Therefore, exploring alternative methods to enable materials to reach this limit more easily is a promising research direction. By laser-processing the PI intermediate layer, we have increased the surface charge density of the TENG’s triboelectric layer by 297% under a normal operating frequency of 5 Hz. Additionally, a comparison of their charging properties revealed that LMI-TENG charges capacitors (4.7 and 23.5 μF) approximately three times faster than FI-TENG (Figure 3d,e). Figure 3f shows the dependence of LMI-TENG output voltage and power density on load resistance, ranging from 1 to 100 MΩ. The results indicate that the voltage increases with increasing load resistance, while the output power initially increases and then decreases as the load resistance increases. The maximum power density output of 8.42 W/m^2^ was achieved at a load resistance of 19 MΩ.

**Figure 3:**
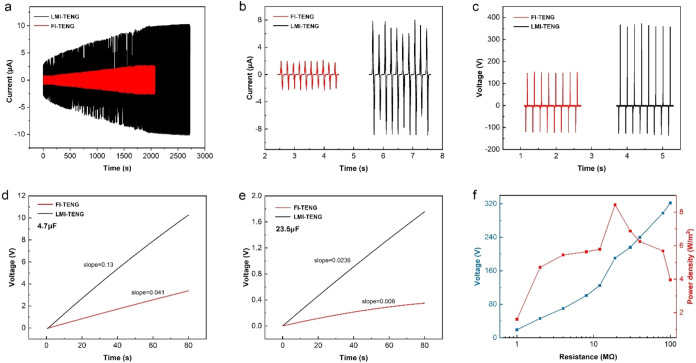
Output performance of TENGs with laser-processed microstructured intermediate layer (black line) and flat intermediate layer (red line): (a) The output accumulation process. (b) The output current. (c) The open-circuit voltage. The charging characteristics with 4.7 μF (d) and 23.5 μF (e) capacitors. (f) The load characteristic of LMI-TENG. (PI intermediate layer thickness of the TENG is 15 μm, and it is processed by a nanosecond laser with 0.28 W and 240 mm/s. The data is measured after 30 min of output accumulation under the impact of 20 N impact pressure and 5 Hz frequency).

### 3.2 Effect of Laser Parameters on Output Performance

To further investigate the role of the laser-processed PI intermediate layer in enhancing the performance of the TENG, we analyzed the mechanisms by which process parameters improve TENG output. Key factors affecting the intermediate layer fabrication include laser power, wavelength, pulse frequency, scanning speed, defocus amount, and spot size. Among these, pulse frequency directly impacts the interval between pulses. Adjusting the frequency is tantamount to simultaneously adjusting the line speed and power. To isolate a single variable for comparison, we fixed the pulse frequency at 200 kHz and the line speed at 200 mm/s. A 355 nm ultraviolet light laser was selected due to its higher single-photon energy and higher material absorption rate, which minimizes thermal effects. Considering the broad range of laser power, a spin coating speed of 900 rpm was chosen to ensure the PVDF triboelectric layer could fully adapt to the structural depth. To prevent excessive deformation and ablation breakthrough, a 100 μm thick PI film was selected, and linear patterns were fabricated to explore the effect of laser power on TENG output. As shown in Figure 4a, when the laser power ranged from 0.1 to 0.22 W, the LMI-TENG output was slightly lower than that of FI-TENG. When the laser power exceeded 1.5 W, LMI-TENG output decreased sharply with increasing laser power. Notably, LMI-TENGs with laser power between 0.22 and 1.5 W all exhibited enhanced output compared to the FI-TENG. The highest performance was observed at 0.8 W, where the fabricated LMI-TENG achieved an open-circuit voltage of 154 V, marking a 220% increase over FI-TENG. Therefore, it can be seen that the laser power has a significant impact on TENG output, which is likely due to the different reactions between the laser and PI caused by the varying single-pulse energy of the laser at different power levels.

**Figure 4:**
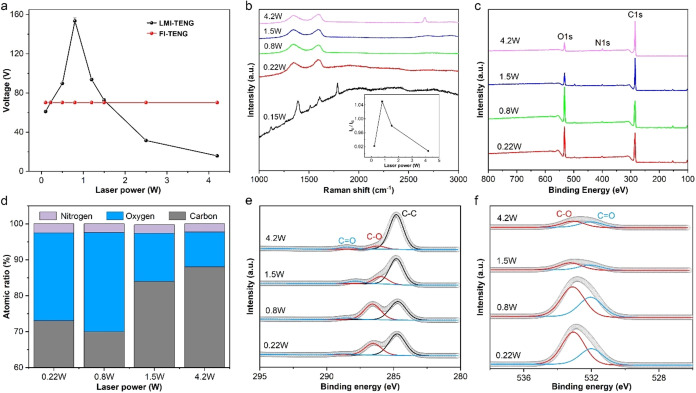
(a) Open-circuit voltage of the LMI-TENG with different laser power, (b) Raman spectra of the PI processed under different laser power; (c–f) XPS spectra of the PI processed under different laser power.

To analyze the surface byproducts formed on PI after laser processing, we performed Raman spectroscopy. As shown in Figure 4b, no carbonization or graphitization of PI occurs at power levels below 0.15 W, with the Raman spectra exhibiting baseline drift or being overshadowed by fluorescence intensity. At a power of 0.22 W, the D and G peaks emerge, indicating the onset of graphitization in PI, but a baseline drift after a 2000-step length suggests that the surface graphitization is only partial. Superdepth field observation suggests that carbonization of another portion of the PI surface contributes to this baseline drift. When the power reaches 0.8 W, the D and G peaks become more pronounced, and a faint 2D′ peak can be seen, indicating an increase in the thickness of the graphite layer on the PI surface as confirmed by electron microscopy. The absence of a prominent 2D peak suggests that the graphite has oxidized. With further increasing laser power, faint 2D and 2D′ peaks are observed at 1.5 W, indicating the formation of a small amount of graphene. At 4.2 W, the D, G, and 2D peaks are distinct. The 2D peak, centered at 2670 cm^–1^ with an FWHM of approximately 65 cm^–1^, suggests the presence of multilayer graphene.^33^ Raman spectra without a 2D peak or with a broadened and low-intensity 2D peak result from the introduction of oxygen-containing functional groups such as hydroxyl (−OH), epoxide (C–O), carbonyl (C=O), and carboxyl (O=C–OH), which disrupt the hexagonal lattice of laser-induced graphene (LIG), leading to structural disorder. Comparison of Raman spectra at five different power levels reveals that at 0.8 W, the laser-treated surface has the highest concentration of oxygen-containing functional groups. Previous reports have indicated that oxygen-containing functional groups such as carbonyl, epoxide, and hydroxyl tend to absorb electrons, thereby endowing the graphite layers with a higher work function.^34^ The half-widths of the D and G bands and the *I*_D_/*I*_G_ ratio reflect the degree of defects and disorder in the graphite. As power increases, the half-widths of the D and G bands gradually decrease, indicating a transition toward more ordered graphite layers. The *I*_D_/*I*_G_ ratio from Figure 4b shows that below 0.8 W, the ratio increases with power, indicating an increase in graphite defects. Above 0.8 W, the *I*_D_/*I*_G_ ratio decreases, suggesting that the increase in power reduces the defects in graphite, leading to a more ordered structure.

Raman spectroscopy has difficulty directly reflecting changes in oxygen elements, yet oxygen is related to the number of defects in the graphite layer.^35^ To further investigate oxygen-related changes, we performed XPS analysis on the graphite layers produced at different laser power levels. As shown in Figure 4c, all four power levels exhibit very high C 1s spectra, with stronger O 1s spectra at 0.22 and 0.8 W. Peaks for the 1s core levels of C, O, and N indicate that the contents of C and O have changed, while the change in N content is not significant, as it has been largely removed from the PI by the laser. The elemental compositions at each power measured by XPS are shown in Figure 4d. The results indicate that with the increase in laser power, the content of oxygen in the graphite layer first increases and then decreases, and the products generated in the power range of 0.22 to 1.5 W can be classified as graphite oxide. At 0.8 W, the content of O reaches a peak of 27.6%, and the O/C ratio is 0.39, which is higher than the original PI. This suggests that O_2_ precipitated from the laser thermal effect layer and the O_2_ component in the air participated in the photothermal reaction of the laser with PI. The content of N only slightly decreases with the increase in laser power, and the N/C ratio is basically maintained at 0.03. The high-resolution C 1s core level spectrum (Figure 4e) is deconvoluted into three main functional groups: C–C (284.7 ± 0.1 eV), C–O (286.4 ± 0.2 eV), and C=O (288.5 ± 0.2 eV). The binding energies for the O 1s core level spectrum (Figure 4f) are respectively C=O (532.1 ± 0.1 eV) and C–O (533.2 ± 0.1 eV). Observing the changes in the core level spectra, we find that the XPS spectra at all four powers show the main C–C peak, while the C–O and C=O vary significantly with the laser power. At the low power of 0.22 W, oxidation is obvious, with higher C–O and C=O peaks observed in both the O 1s and C 1s spectra. As the power increases, the highest C–O and C=O peaks are observed at 0.8 W, where the degree of oxidation caused by laser processing reaches its maximum. With further increase in power, the intensities of the C–O and C=O peaks decrease, and the oxygen-containing functional groups are reduced, which is also corroborated by the results of the Raman spectroscopy.

Combining surface material analysis of the PI film with the surface and cross-section characterization using a superdepth-of-field microscope (Figure S2) and SEM (Figure 5(a–e)), the nanosecond laser processing of PI can be categorized into four stages. At power levels less than 0.1 W, the laser does not react with the PI, with almost all light being transmitted or reflected. Between 0.1 and 0.22 W, the PI primarily undergoes thermal melting and sublimation, with no graphite formation on the surface, but with obvious recondensed structures. Between 0.22 and 0.8 W, the PI surface is irradiated by the laser, with part of it carbonizing and the other part melting due to heat. The gases from carbonization decomposition and the sublimated PI react with the laser to generate a plasma plume. This plasma plume impacts a portion of the melted PI, causing droplets to be sputtered. The sputtered PI droplets then react under photothermal conditions to form graphite oxide, which then falls back onto the PI surface under the influence of gravity and cools and solidifies. When the laser power approaches 0.8 W, the single pulse energy approaches the photothermal reaction threshold of PI, resulting in a uniformly dispersed graphite oxide layer with moderate thickness, thus maximizing TENG output. The graphite oxide formed between 0.22 and 0.8 W is considered to be indirectly generated, with an increase in power leading to an increase in the thickness of the graphite oxide layer on the PI surface. The thermal effect layer and the graphite oxide layer on the surface enhance TENG output. When the power is between 0.8 and 1.5 W, the graphite oxide layer induced on the PI surface by the laser continues to thicken with increasing power and exhibits a regular flaky pattern. Once the laser power exceeds 1.5 W, the PI surface begins to directly generate porous graphite oxide with a thickness exceeding the depth of the structure, causing a sharp decrease in TENG output. Continuing to increase the laser power, vertical graphene starts to form on the PI surface, and with the continued increase in power, the volatiles in the thermal effect layer increase, leading to an increase in the thickness and pore structure of the surface-formed graphene. When the power exceeds 4.2 W, the PI film exhibits ablation penetration and cannot be used for subsequent fabrication.

**Figure 5:**
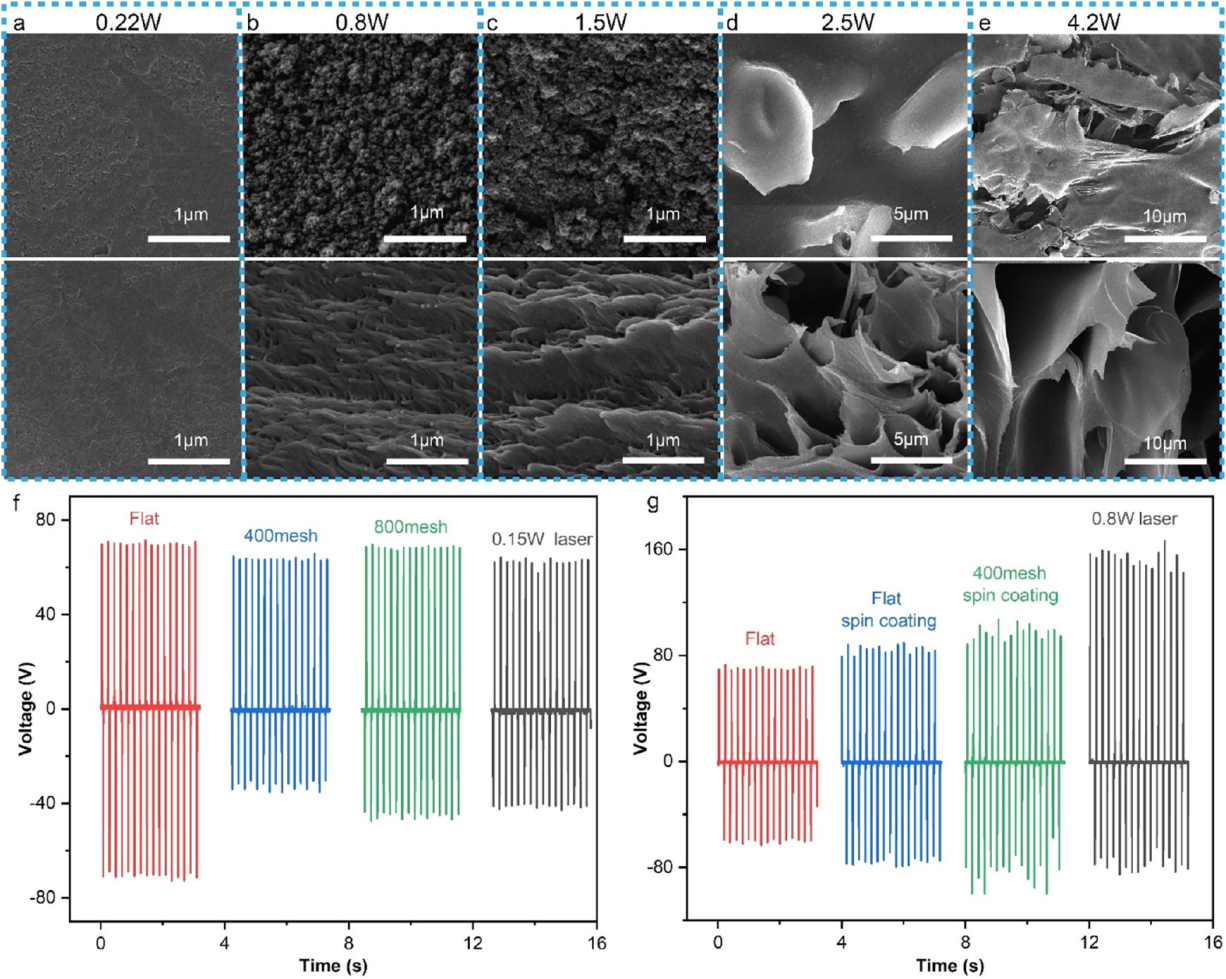
(a–e) SEM images of the laser processed PI intermediate layer under different power levels, with the upper side being the surface and the lower side being the cross-section. (f) The output voltage of TENGs fabricated by different methods for the intermediate layer structure; (g) the output voltage of TENGs fabricated by different methods for the surface graphene oxide layer of the intermediate layer. (The thickness of the PI intermediate layer of TENG is 100 μm, and the data is measured after being impacted for 30 min at an impact pressure of 20 N and a frequency of 5 Hz).

Previous studies have proven^36,37^ that increasing the dielectric constant of the medium is an effective strategy to enhance TENG output, such as by forming a conductive intermediate layer within the dielectric base. The integration of conductive fillers into the insulating layer facilitates electron trapping, forming microcapacitors that significantly improve the air breakdown limit and surface charge density. The graphite oxide layer obtained by nanosecond laser processing of PI acts as a conductive medium, forming in sync with the processing structure and adhering to the PI surface. Due to these processing characteristics, the dispersed graphite oxide between the middle and triboelectric layers functions as capacitors on the PI surface, capturing and storing charge to enhance output. Additionally, the graphite oxide layer between the triboelectric layer and the intermediate layer also provides a charge transfer channel, allowing newly generated charges in the triboelectric layer to migrate more efficiently into the shallow traps in the PI’s thermal effect layer,^38,39^ resulting in deeper charge trapping within the PI.^40^ When the power reaches 0.8 W, the number of oxygen vacancy defects in the graphite oxide layer peaks, enhancing electron absorption and enabling PI to store charge more efficiently. Within the 0.8 to 1.5 W range, as power increases, oxygen content in the graphite oxide layer decreases while conductivity improves, making it easier for charges generated by the triboelectric layer to dissipate into the air, leading to a lower surface charge density and a reduction in the increase of output voltage. In the power range of 1.5 to 4.2 W, the graphene produced has even stronger conductivity, exacerbating the neutralization and drift of triboelectric charges, causing leakage when exceeding the percolation threshold, resulting in a sharp drop in the surface charge density of the triboelectric layer and TENG output. At the same time, the increased thickness of the graphene, acting as a conductor, can shield the electric field between the triboelectric layer and the electrode, also leading to a decrease in output voltage. Due to the accumulation of heat, the greater the power, the larger the deformation of the PI film after processing, resulting in a lower yield of functional TENGs. Correspondingly, the spin-coated PVDF surface also becomes uneven due to the significant height difference in the graphene, leading to a reduction in the contact-separation area between the triboelectric layers, which is another reason for the reduced output when the laser power is in the range of 1.5 to 4.2 W. When laser power is below 0.22 W, processing affects only the surface structure and its associated thermal effect layer. To differentiate the impact of structure and the thermal effect layer on output, a control experiment was conducted by sanding a 100 μm thick PI film to make a sanded intermediate layer TENG, which was compared with LMI-TENG. The sanded intermediate layer TENG exhibited reduced output compared to the FI-TENG (Figure 5f), indicating that merely fabricating structures on the intermediate layer does not enhance output, aligning with previous findings.^11^ The output reduction was attributed to poor contact between the spin-coated PVDF triboelectric layer and the PI intermediate layer, likely caused by mismatched spinning speed or solution concentration. Additionally, the LMI-TENG with power less than 0.22 W and the sanded intermediate layer TENG yielded similar outputs, suggesting that at power levels below 0.22 W, the impact of laser irradiation and thermal accumulation on PI surface structure is minimal. For power levels between 0.22 and 0.8 W, the key factor affecting TENG output is the graphite oxide layer produced by laser processing. To evaluate the impact of the graphite oxide byproducts on TENG output, output tests of TENGs with graphite oxide layers prepared on the PI intermediate layer by different methods were designed. As shown in Figure 5g, the output of the TENG with the laser-processed graphite oxide layer increased by 56% compared to the TENG with the spin-coated graphite oxide layer after sanding, and increased by 220% compared to the FI-TENG. Moreover, the TENG with the spin-coated graphite layer after sanding increased output by 21% compared to the TENG with a flat spin-coated graphite layer. These findings indicate that a graphite oxide layer of appropriate thickness can directly serve as a charge transfer layer to enhance TENG output. Additionally, increased charge transfer efficiency caused by a larger contact area between the graphite oxide conductive layer and the triboelectric layer further boosts output, which is consistent with the experimental results of the power analysis. Overall, these comparisons demonstrate that laser processing offers distinct advantages over direct coating in enhancing TENG performance. The observed improvements are not solely due to the graphite oxide layer but also stem from the laser thermal effect layer, which should be considered in optimizations.

To investigate the impact of the laser-induced thermal effect layer on TENG output, we conducted EDS elemental analysis on the cross-section of the thermal effect zone of the laser-processed TENG. The selected test points are distributed on the thermal effect layer as shown in Figure 6a, where point 1 represents the upper thermal effect layer, point 2 represents the middle thermal effect layer, and point 3 represents the lower thermal effect layer. Referring to the research by Inagaki, between 500 and 650 °C, the partial breakage of carbonyl groups in polyimide leads to the sudden release of CO and CO_2_, and between 800 and 1000 °C, polyimide thermally decomposes to produce H_2_, O_2_, and N_2_.^41,42^ When the laser power is less than 0.22 W, the elemental changes of C, N, and O in the cross-section are minimal, as seen in Figure 6(b–d), and there is no deformation of the PI film, with almost no carbonization on the surface. This indicates that the temperature of the laser thermal effect layer is below 650 °C, and the minor release of C and O elements has little overall impact. When the laser power exceeds 0.22 W, rapid thermal accumulation caused by the laser quickly raises the temperature of the thermal effect layer above 800 °C, leading to the release of elements such as N and O from the polyimide. When the laser power reaches 0.8 W, the polyimide’s laser thermal effect layer is at the initial stage of carbonization, and the content of N and O elements gradually increases from the upper to the lower side of the thermal effect layer. The upper side of the thermal effect layer, being closer to the laser processing area, accumulates heat faster, causing thermal breakage of C–N–C and C=O bonds in the PI to form N_2_ and O_2_, increasing defects in the upper thermal effect layer. As a result, the number of shallow traps in the PI’s laser thermal effect layer increases, capturing more charges. As the laser power increases, the content of oxygen and nitrogen elements in the thermal effect layer decreases, while the proportion of carbon increases. When the laser power exceeds 1.5 W, the carbon content in the upper side of the thermal effect layer near the graphite oxide layer exceeds 90%. Combined with the analysis of the graphite oxide layer, excessive carbonization of the thermal effect layer causes C elements to bond, and the number of shallow traps decreases accordingly. The reduction in charges captured and conducted by shallow traps leads to a decrease in the amount and storage of charges captured by deep traps in the PI, causing a further decrease in TENG output.

**Figure 6:**
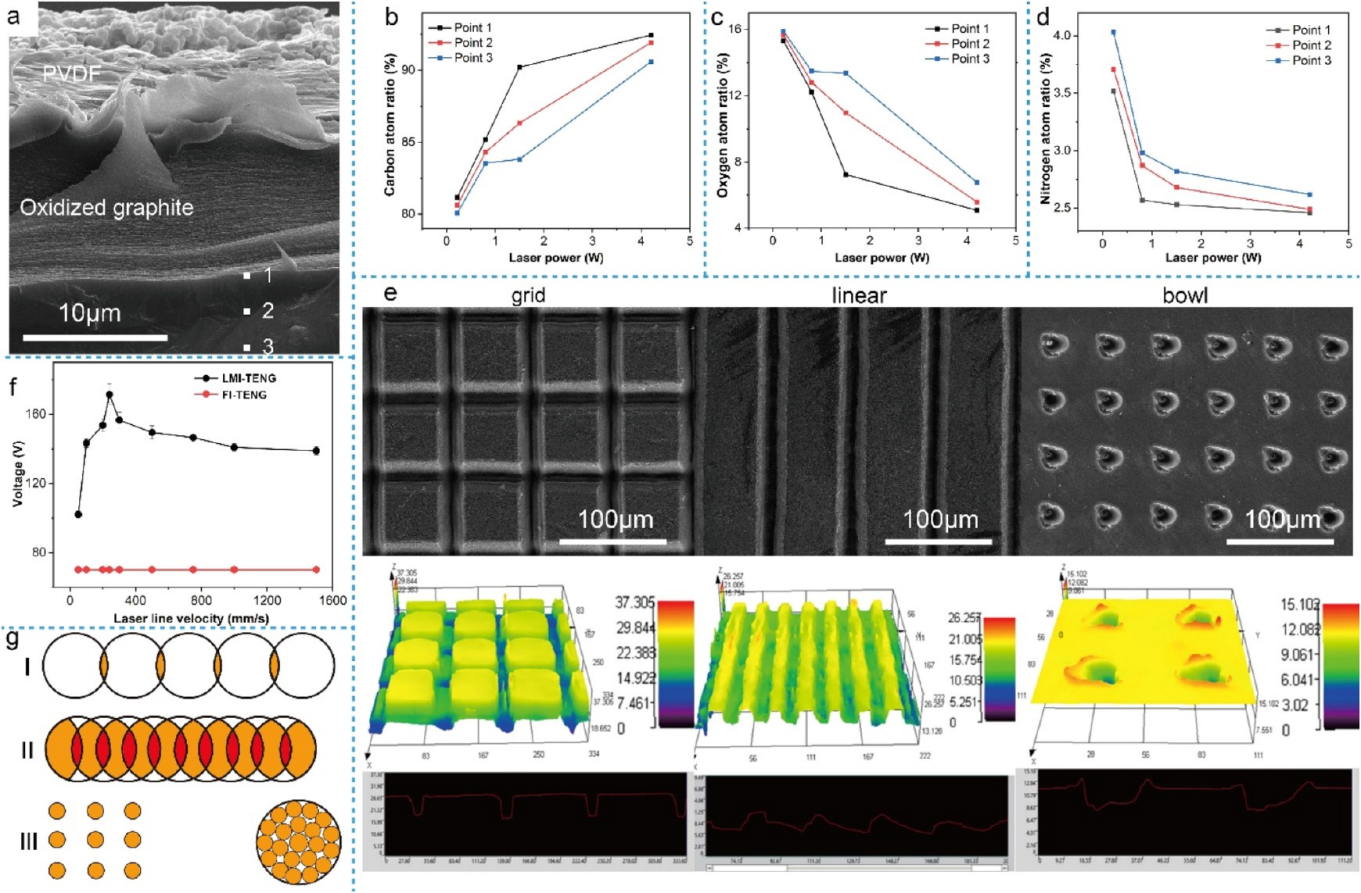
(a) Cross-sectional image of the LMI-TENG and the selection of EDS test points. (b) The change of C content with the laser power. (c) The change of O content with the laser power. (d) The change of N content with the laser power. (e) SEM and ultradepth-of-field surface images of the reticular, linear, and bowl-shaped patterns prepared by nanosecond laser at 0.8 W. (f) Output voltage of LMI-TENG (the laser power is 0.8 W, and the output voltage is measured after being impacted for 30 min at an impact pressure of 20 N and a frequency of 5 Hz). (g) The process method and pulse repetition rate of laser processing.

Beyond laser power, the processing method and laser pattern also significantly impact TENG output. Using the optimal nanosecond laser power of 0.8 W, we fabricated mesh, linear, and bowl-shaped microstructures on a 100 μm-thick PI intermediate layer and compared their respective TENG outputs. All three patterns improved output compared to FI-TENGs, with the linear structure exhibiting the highest increase (Figure S3a). In Figure 6e, it can be seen that the thickness distribution of the graphite oxide layer formed on the surface of the mesh and bowl-shaped structures is uneven, which greatly affects TENG output. For the linear pattern, the thicker parts underwent multiple scans, with the initial scan partially oxidizing the surface and accumulating heat on the PI surface. Due to the high speed of laser processing, the energy of subsequent scans plus the heat accumulated previously reaches the threshold of the photothermal reaction, which makes graphene oxide generated in situ at the intersection points of the lines scanned many times (Figure S3b). The in situ formed graphene oxide has a reduced oxygen content and fewer defects, which decreases its ability to store charge, leading to a reduction in TENG output. Additionally, under the condition of a fixed single pulse energy, the line speed and processing route also affect the output. The processing of linear patterns is greatly affected by the line speed and processing interval. Figure 6f shows the influence of laser processing line speed on the output when processing linear patterns, with supplementary images of the processing morphology in super depth of field shown in Figure S3c. When the line speed is 150–300 mm/s, the processing morphology is optimal, and further increasing or decreasing the line speed worsens the morphology. At speeds greater than 1000 mm/s, the pulse morphology of the laser processing can be seen in the linear structure. At speeds below 50 mm/s, due to heat accumulation, the PI film becomes scorched and carbonized, and the deformation of the film after processing is too great for subsequent TENG fabrication. As the laser line speed decreases, the processing depth increases, and the output first increases and then decreases. At a line speed of 240 mm/s, the TENG achieves the maximum output, with an open-circuit voltage 11% higher than at 200 mm/s and 133% higher than the TENG without any structure. The line speed of laser processing mainly affects the processing depth and morphology, with the morphology having little impact on the output, which is mainly determined by the processing products and depth. As shown in Figure 6g­(I), increasing line speed results in wider pulse spacing and a lower repetition rate, producing more uneven surface products. A decrease in laser line speed increases the repetition rate, which makes the surface products more uniform. However, when the line speed is too low, the probability of pulse overlap increases, again leading to an uneven surface structure (Figure 6g­(II)). This analysis, combined with previous findings on the graphite oxide layer, confirms that a uniformly oxidized graphite layer plays a key role in enhancing TENG output. Therefore, the impact of laser line speed on output is relatively small because the line speed does not affect the energy of individual laser pulses. Instead, it mainly affects the pulse overlap rate at the same processing location, which manifests as processing depth and surface morphology in the fabrication process. A decrease in line speed increases the processing depth, which effectively thins the PI film and optimizes the thickness of the intermediate layer, leading to an enhanced output. For bowl-shaped structures, Huang et al.^26^ used a single-pulse processing method, where one pulse fabricates one bowl-shaped structure. This method requires a relatively high line speed, and the processing effects are greatly influenced by parameters such as light spot size, defocus amount, and energy distribution at the focal point. Among these, the energy distribution at the focal point is particularly critical, as it affects bowl shape formation and surface product characteristics, ultimately influencing TENG output. While single-pulse processing is suitable for rapid fabrication of small structures less than 10 μm in size multipulse processing is more suitable for larger structures requiring uniform energy distribution, as shown in Figure 6g­(III). By adjusting the energy distribution at the focal point and selecting the pulse processing method, morphologies of different scales can be fabricated rapidly, and the distribution of graphite generated during processing can be made more uniform. This increases the range of triboelectric layer thicknesses compatible with the intermediate layer structure, and the TENG output can be improved by optimizing the total dielectric layer thickness (Figure S4). Therefore, for laser parameters, there is an optimal line speed and best processing method that maximize TENG output.

### 3.3 Influence of Laser Processing on Output Characteristics and Cycle Life

The processing of structures on the PI intermediate layer also significantly influences TENG output characteristics. When there are structures on the intermediate layer, gaps are likely to form between the intermediate layer and the triboelectric layer after spin-coating (Figure S5a). Additionally, large height differences in these structures can result in an uneven PVDF surface (Figure S5b). Both scenarios can cause additional or sequential contact between the two triboelectric layers during operation, resulting in a multipeak voltage output. As shown in Figure 7a, the LMI-TENG exhibits the most multipeak voltages, followed by the TENG with the sandpaper-treated intermediate layer, while the FI-TENG shows only a few multipeaks. Figure 7b illustrates that the FI-TENG has a narrow single-peak pulse width during output, while the LMI-TENG has a high proportion of multipeaks with a larger pulse width. Sequential contact can lead to a reduction in the maximum output voltage generated when the copper electrode and PVDF triboelectric layer make contact but can also cause additional separation of triboelectric charges and increase the total output charge. Consequently, this leads to an elevation in the maximum output voltage during separation.

**Figure 7:**
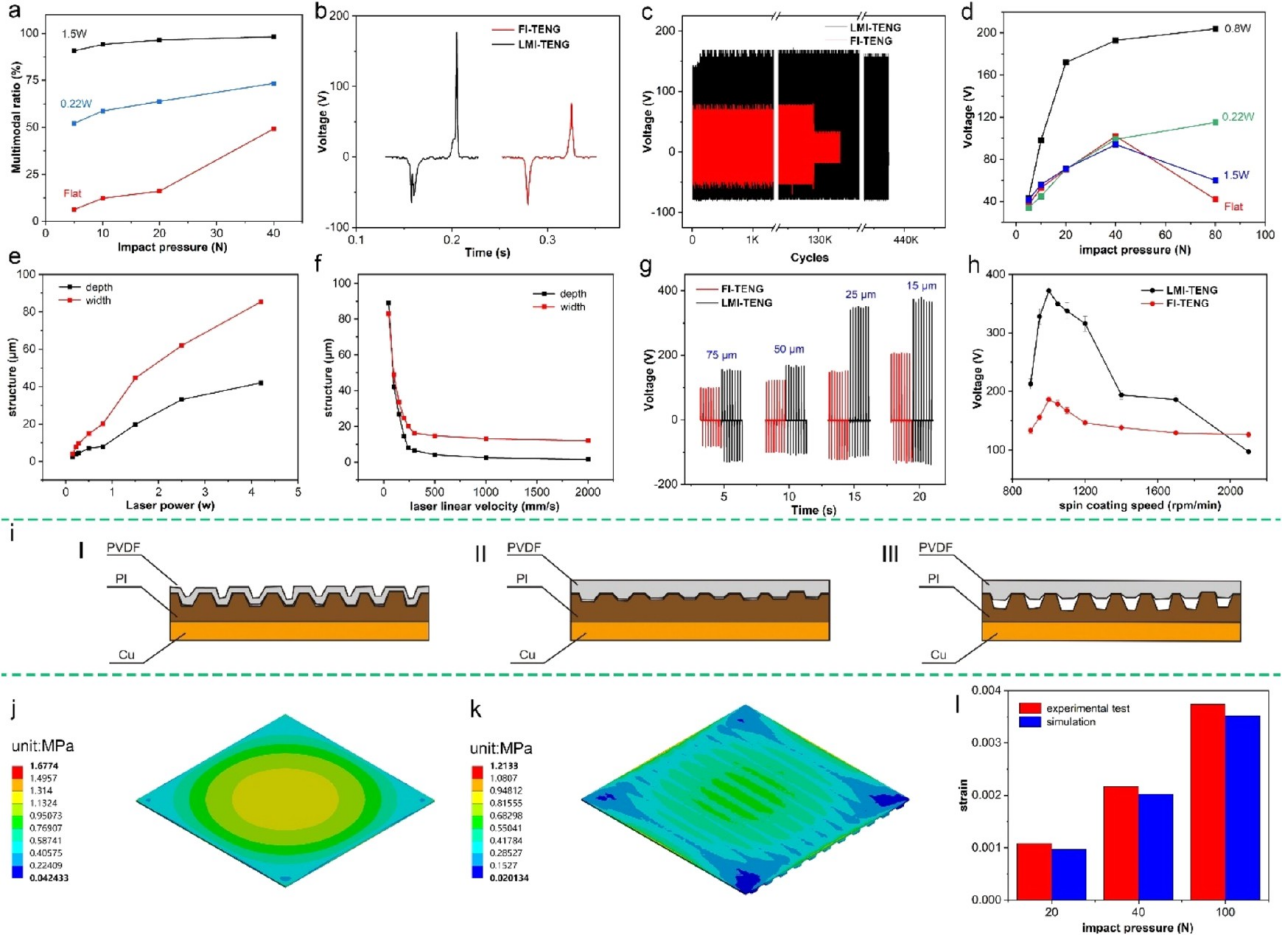
(a) Multipeak ratios of FI-TENG and LMI-TENG with node power under different pressures. (b) Voltage output of the LMI-TENG. (c) Cyclic life of the TENG. (d) The impact pressure and voltage output. (e) The relationship between the laser power and the processing depth and width (laser linear velocity is 240 mm/s). (f) The relationship between the laser linear velocity and the processing depth and width (laser power is 0.8 W). (g) Output voltage of the TENG prepared by laser processing different thickness intermediate layers. (h) The relationship between the PVDF spin-coating speed measured on the 15 μm PI and the output of the LMI-TENG. (i) The bonding mode of the spin-coated PVDF layer and the laser-processed PI intermediate layer. (j) The equivalent stress of the PVDF layer in the FI-TENG. (k) The equivalent stress of the PVDF layer in LMI-TENG with well bonded. (l) The strain results of the PVDF layer in the LMI-TENG with well bonded under different pressures. (The data in (a, b, g, and h) were measured after 30 min of cycling at a frequency of 5 Hz and an impact pressure of 20 N, and (d) was measured after 30 min of cycling at a frequency of 5 Hz. (c) was measured at a frequency of 5 Hz and an impact pressure of 20 N).

The cycle life of TENGs is crucial for practical applications, and laser processing significantly improves the cycle life of TENGs. As shown in Figure 7c, the output voltage of the FI-TENG decreases after approximately 150,000 cycles of operation, while the LMI-TENG can operate for about 280,000 more cycles. However, after 430,000 cycles, the output of the LMI-TENG decreases, and the reduction in output for both is due to the delamination of the PVDF triboelectric layer. Moreover, since the byproducts of laser processing are in situ grown and flexible, the device can maintain its integrity well during various mechanical deformations. Compared to a directly applied graphite oxide layer, the laser-processed graphite oxide layer has stronger bonding with PVDF and PI, making it more resistant to damage or detachment during impacts or deformations. Due to the influence of the microstructures in the intermediate layer, the thickness of the spin-coated PVDF is uneven, and the stress is more dispersed when subjected to impact, resulting in a 286% increase in the cycle life of the LMI-TENG compared to FI-TENG. The triboelectric layer of the LMI-TENG has a larger bonding area and higher strength with the intermediate layer, making it less prone to delamination and able to withstand greater impact forces (Figure 7d). Although greater impact generally enhances output, excessive pressure can lead to triboelectric or intermediate layer damage. The FI-TENG experiences damage to the PVDF triboelectric layer under the impact of 80N pressure, while the reduction in output for the TENG with 1.5W laser processing under the same conditions is due to the collapse and detachment of the graphite oxide layer.

### 3.4 Influence of Dielectric Thickness on TENG Output

Research indicates that the total dielectric thickness (*d*_1_) significantly affects TENG output. Zhang^43^ identified an optimal dielectric thickness that can enhance output by optimizing both the gap and dielectric layer thickness. The total dielectric thickness includes the triboelectric layer and the intermediate layer. The thickness of the triboelectric layer mainly determines the electron generation rate, while the intermediate layer primarily reduces charge decay in the triboelectric layer by storing charges, thereby increasing the charge quantity in the dielectric layer. Therefore, we first processed linear structures on PI intermediate layers with different thicknesses to determine the optimal intermediate layer thickness for enhancing output. TENGs were fabricated using nanosecond laser processed PI intermediate layers of varying thicknesses (15, 25, 50 and 75 μm). Figure 7e,7f demonstrate the relationship between laser power, line speed, and the resulting processing depth and width, indicating that laser power significantly affects the processing width, while line speed has a more pronounced impact on processing depth. To adapt the structure and thickness and to unify experimental variables, the processing depth was kept below 15 μm, selecting a laser power of 0.28 W and a line speed of 240 mm/s with a line spacing of 12 μm. The resulting output voltages (Figure 7g) confirm that laser processing enhances TENG output across different intermediate layer thicknesses. Specifically, at 25 μm PI thickness, the LMI-TENG demonstrated a 233% increase in open-circuit voltage, while at 15 μm, the TENG achieved a maximum open-circuit voltage of 370 V. These results suggest that one reason for the enhanced output due to laser processing is the reduction in intermediate layer thickness, and the closer it is to the optimal intermediate layer thickness, the less the output is improved by this aspect. This is corroborated by the increase in output voltage for the 15 and 25 μm layers shown in Figure 7g. Generally, a thinner triboelectric layer is considered beneficial for enhancing the capacitive characteristics and electrostatic induction of TENGs but is not favorable for the generation of triboelectric charges. A thicker triboelectric layer will generate more triboelectric charges, but more intrinsic carriers in PVDF can also promote the rapid neutralization of triboelectric charges, reducing surface charge density. Based on this, to optimize the PVDF triboelectric layer thickness to match the laser-processed microstructures on the PI intermediate layer and boost TENG output, we controlled the spin-coating speed to adjust the bonding between the PVDF triboelectric layer and the PI intermediate layer. As shown in Figure 7h, PVDF was spin-coated onto laser-processed linear structures on a 15 μm PI film. Increasing spin speed initially improved performance before causing a decline. The relationship between triboelectric layer thickness and intermediate layer structure can be categorized into three bonding modes: when the thickness of the triboelectric layer is less than the depth of the intermediate layer’s structure and is tightly bonded to it, the uneven surface of the triboelectric layer results in reduced contact area between the two triboelectric layers, and the output decreases sharply (Figure 7i­(I)). Furthermore, a thinner triboelectric layer necessitates high-speed spin-coating, which can result in a decline in film quality, potential small holes, and uneven thickness due to secondary acceleration, all of which can diminish cycle life and output. When the spin-coating speed is 1000 rpm, the PVDF forms a good film and tightly bonds with the PI, yielding the best output performance for the laser-processed intermediate layer TENG (SEM cross-section provided in Figure S5c). At this point, the bottom surface of the PVDF triboelectric layer attains the maximum bonding area with the PI intermediate layer, and the flatness of the PVDF’s upper surface also ensures a stable contact area between the two triboelectric layers (Figure 7i­(II)). When the PVDF friction surface is smooth but poorly bonded to the PI intermediate layer, that is, the contact area is stable but the bonding area is reduced (Figure 7i­(III)), this will also lower the TENG output. This typically occurs when the intermediate layer structure is mismatched with the solution concentration or spin-coating speed. Meanwhile, due to the structural depth and thermal stress from laser-processed PI, poor bonding easily occurs when spin-coating PVDF on the microstructured PI surface (Figure S6), which also reduces the output of LMI-TENG.

By combining the previous analysis of the TENG cycle life with three bonding modes of PVDF/intermediate layer, we believe that laser processing alters stress distribution in the PVDF/PI layer, influencing TENG durability. To verify this, we used Ansys simulations to analyze stress distribution under impact pressure. As shown in Figure 7j,7k, in LMI-TENGs with good bonding, the equivalent stress in the central region of the PVDF layer is 38.2% lower than that in the FI-TENG due to the presence of microstructures, which increase the interlayer contact area. Furthermore, stress probe analysis reveals that laser processing shifts the maximum stress point from the PVDF layer edges (in FI-TENGs) to the sides of the PVDF layer (in LMI-TENGs). This shift enhances the reliability of the PVDF-intermediate layer interface, improving mechanical robustness. Although the reduction in internal stress affects the charge generation rate, the output of the well-bonded LMI-TENG is still significantly improved compared with that of the FI-TENG, which further highlights the enhancement of laser processing on the charge transfer/storage performance of the PI intermediate layer. When the PVDF layer becomes uneven on the surface due to the reasons in Figure 7i, the contact area between the triboelectric layers will decrease, and the equivalent stresses in the PVDF layer and the graphite layer will both increase. This situation can be described by eq 3.

3 $$\sigma =\frac{F}{A}$$

Where σ is the stress, *F* is the acting force, and *A* is acting area.

Generally, an increase in pressure enhances the charge generation rate, but the output of TENG decreases when the triboelectric layer and intermediate layer are poorly bonded. This indicates that poor bonding reduces the efficiency of charge transfer between the triboelectric layer and intermediate layer, causing most of the generated charges to drift and neutralize within the triboelectric layer. As shown in Figure S7a, when the laser power is relatively low, the surface stress of the PVDF layer increases by 272% compared to that of the FI-TENG due to the undulations caused by the excessively thin spin coating layer. When the laser power is relatively high, the graphene generated on the PI surface has excessive undulations, which causes the spin coating within the subsequent parameter range to lead to undulations in the PVDF layer. This will further reduce the contact area of the triboelectric layer of the LMI-TENG, and the reduction in the contact area causes the surface stress of the PVDF layer to increase sharply, reaching 4.57 MPa (Figure S7b). Figure S7­(c–f) shows the stress simulation on the intermediate layer/graphite layer. It can be found that the stress on the intermediate layer of the LMI-TENG with good bonding is reduced by 34.7% compared to the FI-TENG. This reduction is attributed to the graphite oxide layer’s ability to absorb impact energy, as well as its relatively high Young’s modulus. Moreover, the stress on all intermediate layers/graphite layers is greater than that on the PVDF layer, and both the stress concentration and the maximum stress point are located in the middle part. This also well confirms why failures often occur in the middle part during fatigue testing. According to the general principles of material fatigue, the larger the cyclic stress, the shorter the fatigue failure cycle. When the LMI-TENG has good bonding, it maximizes the output, and at this time, the equivalent stress of the PVDF layer and the graphite layer is reduced, thereby increasing the cycle life of the TENG. Hooke’s law indicates that within a certain proportional limit, there is a linear proportional relationship between stress and strain. To verify the accuracy of the simulation, we used a digital display dynamometer and a resistive strain sensor to conduct a strain test on the TENG and compared it with the simulated equivalent strain (Figure S7h,g). The results are shown in Figure 7l. The strain errors of the experiment and simulation under different pressures are all less than 8.6%, which indicates the credibility of the stress simulation. Therefore, for the LMI-TENG, in addition to maximizing the bonding area between the triboelectric layer and the intermediate layer as much as possible, the total medium thickness, the optimal thickness of a single medium, and the matching of the thicknesses of various media should also be considered to ensure a stable contact area between triboelectric layers and reduce the stress of the triboelectric layer and the intermediate layer, thereby maximizing the output and cycle life improvement.

Laser processing modifies the Young’s modulus and adhesion force of the PI surface, which exerts a profound impact on the service life of the TENG. Among these factors, the Young’s modulus is primarily influenced by the surface composition and structure. It is generally believed that Young’s modulus is related to the stability of the surface structure: a higher Young’s modulus means a greater force is required to achieve the same degree of deformation, and more energy is absorbed in the process.^44,45^ As shown in Figure 8a–d, the average Young’s modulus of PI is approximately 1.1 GPa. Laser processing induces the formation of a graphite oxide layer on the PI surface: at a power of 0.22 W, the average Young’s modulus of this graphite oxide layer is 4.8 GPa; at the threshold power of 0.8 W, the formed graphite layer has a relatively low thickness, with an average Young’s modulus of about 11.4 GPa; when the laser power increases to 1.5 W, the average Young’s modulus of the formed porous graphene oxide layer reaches 179.3 GPa. It can be observed that as the laser power increases, the oxygen content of the graphite oxide layer decreases, and the layer gradually transforms into graphene oxide/graphene. Moreover, due to the reduction in oxygen vacancy defects, the Young’s modulus of the layer increases accordingly, which is consistent with Mondal’s research.^46^ Since 0.22 W is the threshold power for graphite oxide formation, the photothermal reaction of PI at this power is unstable, resulting in a relatively large laser heat-affected zone (HAZ). We also measured the Young’s modulus of the laser HAZ and found that the Young’s modulus of the cross-sectional HAZ varies significantly (Figure 8j), with an average value of 23.6 GPa—much higher than that of the graphite layer (Figure 8e). Referring to Méndez’s research^47^ and our previous analysis of the carbon–oxygen ratio in the laser HAZ, we attribute this significant variation in Young’s modulus to the increased crystallinity of the polymer caused by recrystallization-induced carbonization of PI, as well as the incorporation of graphite oxide into some regions during recrystallization. In addition, we measured the Young’s modulus of the cross sections of PI graphite oxide layers processed at 0.8 and 1.5 W, and found that the Young’s modulus of the cross sections is lower than that of the surfaces. This indicates the anisotropic mechanical strength of the layers (Figure S8a,b). Figure 8f–i show the bar distributions of the Young’s modulus of the PI surface and the PI intermediate layers processed at threshold powers. It can be seen that as the laser power increases and the graphite oxide layer gradually transforms into graphene, the bar distribution of its Young’s modulus becomes more concentrated, this suggests a reduction in defects and an improvement in the quality of the graphite layer. As shown in Figure S8c–f, we analyzed the surface adhesion of the graphite oxide layers on laser-processed PI surfaces. The results show that the average adhesion of the graphite oxide layer processed at 0.22 W reaches 347 nN, which is 50 times higher than that of PI. We attribute this to the highest oxidation degree achieved at this power, which is also supported by Zeng’s research.^48^ As the laser power increases, the adhesion of the graphite oxide/graphene layers gradually decreases. Adhesion affects the subsequent spin-coating process and the interfacial bonding strength between PVDF and the intermediate layer; an increase in interfacial strength and bonding area facilitates the transfer of loads from the substrate to high-strength materials.^49^ The increase in the Young’s modulus of the intermediate layer and the enhancement of surface adhesion contribute to the improvement in the cycling lifespan and maximum test pressure of the LMI-TENG. However, the large difference in Young’s modulus may also lead to the detachment of PVDF after long-term impact. We supplemented the cycling lifespan of the TENG with a 1.5 W laser-processed intermediate layer in Table S1, and found that it is lower than that of the TENG processed at 0.22 W.

**Figure 8:**
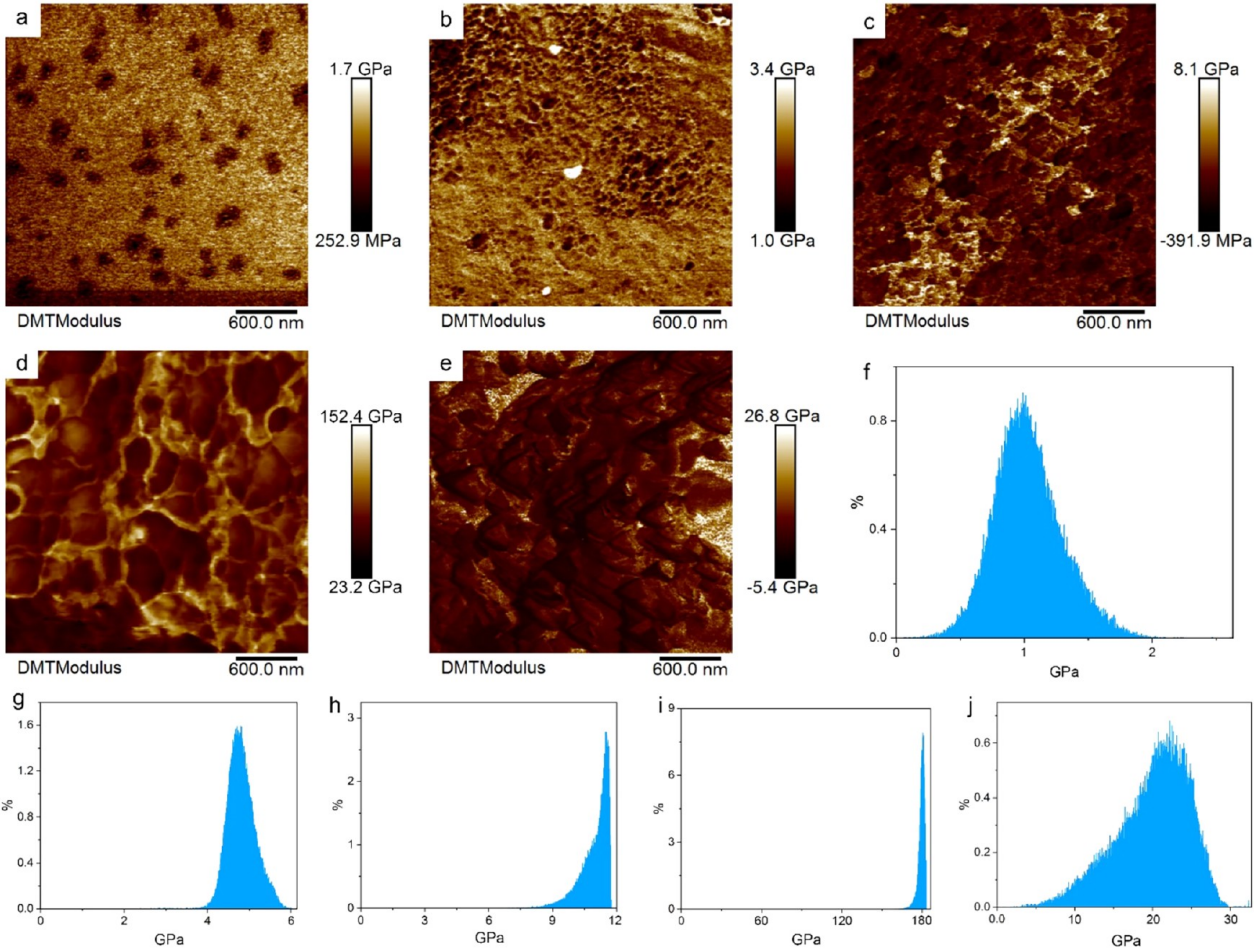
(a–e) show the 2D maps of the surface Young’s modulus of the PI intermediate layer obtained via AFM testing, (f–j) represent the bar charts of Young’s modulus. (a, f) correspond to the PI surface (untreated); (b, g) correspond to the surface processed by 0.22 W laser; (c, h) correspond to the surface processed by 0.8 W laser; (d, i) correspond to the surface processed by 1.5 W laser; (e, j) correspond to the cross-section processed by 0.22 W laser.

From the above research, it can be concluded that laser power is the laser parameter exerting the most significant influence on the output performance and cycle life of the TENG. Adjusting parameters such as linear speed or focal energy distribution is essentially equivalent to regulating the regional laser power. Specifically, the impact of laser power on structural depth alters the contact area between the triboelectric layer and the intermediate layer, which in turn affects the stress distribution. Meanwhile, varying laser powers induce changes in the Young’s modulus of PI intermediate layer surface—this also exerts an influence on the cycle life of the triboelectric layer and intermediate layer under impact conditions. Therefore, in Table S1, we summarize the effects of laser power on the surface composition, structure, elastic modulus of the PI intermediate layer, and the output performance of the TENG, providing a convenient reference for readers to review and analyze the key findings.

Table 1 provides a performance comparison between the TENG prepared in this study and those fabricated by other laser processing methods. Compared with previous studies, this work clearly demonstrates enhanced output performance through ultraviolet nanosecond laser-engineered microstructured intermediate layer, which is reflected in the following aspects: First, the charge accumulation process is revealed. The graphene oxide layer mainly serves to rapidly transport charges from the triboelectric layer and capture/storage charges, thereby facilitating the better transfer of charges to the deep traps in the PI layer. Second, the optimal thicknesses of the dielectric layers, including the triboelectric layer and the intermediate layer, are explored. By matching the charge generation rate of the triboelectric layer with the capture/storage rate, and minimizing the impact of thickness on the electrostatic induction between the dielectric layer and electrodes as much as possible, the output of the TENG is improved. Third, the laser processing parameters for the intermediate layer are explored and analyzed, which is of great significance for large-scale industrial production. Finally, the influence of the laser-processed intermediate layer on the cycle life of the triboelectric nanogenerator is innovatively analyzed.

**Table 1:** Energy Harvesting Performance of TENGs Fabricated by Various Laser Processing Method

method	process	output voltage (V) and current (μA)	power density (W/m^2^)	test pressure (N) and frequency (Hz)	refs
femtosecond laser	pattern the surface of the PDMS triboelectric layer	42.5 V 10.1 μA	1.73 W/m^2^	100 N 3 Hz (2 cm × 2 cm)	^50^
femtosecond laser	pattern both the PDMS and Cu triboelectric layer	22.04 V 2.6 μA	0.21 W/m^2^	2 N 1.5 Hz (8 mm × 8 mm)	^26^
Q-switched pulsed laser	fabricate nanostructures on the surface of the aluminum Triboelectric layer	148 V 9.6 μA	0.14 W/m^2^	10 N 1 Hz (5 cm × 5 cm)	^24^
CO_2_ laser	induce graphene on the carbon precursor as the electrode	3500 V 60 μA	2.4 W/m^2^	2 N 2 Hz (6 cm × 6 cm)	^25^
semiconductor continuous laser	induce graphene on the PI substrate as the electrode	119 V 11 μA	0.61 W/m^2^	10 N 10 Hz	^51^
picosecond laser	induce F-doped graphene electrodes at the interface between FEP and PI	198 V 103 μA	47.5 W/m^2^		^52^
CO_2_ laser	induce graphene on the PI substrate as the and electrode	165 V			^53^
Laser	induce graphene and add MoS_2_ to make it serve as an intermediate layer	31 V	0.047 W/m^2^	3 Hz (3 cm × 3 cm)	^36^
CO_2_ laser	carbonized MXene/ZiF-67 nanocomposite serves as an intermediate layer	1340 V	65 W/m^2^	10 N 4 Hz (2 cm × 2 cm)	^27^
CO_2_ laser	induce graphene to serve as an intermediate layer	126 V 17.5 μA	0.12 W/m^2^	20 N 3 Hz (4 cm × 4 cm)	^54^
nanosecond laser	simultaneously fabricate the microstructure and graphene oxide layer on the PI intermediate layer	370 V 8.4 μA	8.42 W/m^2^	20 N 5 Hz (15 mm × 15 mm)	This work

## 4 Conclusions

In summary, we demonstrated a novel approach to enhance the output performance and durability of TENGs through ultraviolet nanosecond laser-engineered microstructured intermediate layers. By optimizing laser processing parameters, including power, scanning speed, and structural design, we significantly improved charge storage within the intermediate layer, resulting in a notable increase in TENG output. The LMI-TENG achieved a 3.8-fold increase in short-circuit current and a 2.3fold increase in open-circuit voltage compared to the FI-TENG. Specifically, laser processing of linear patterns on a 15 μm-thick PI intermediate layer at 0.28 W and 240 mm/s resulted in a 3-fold increase in the charging rate, enabling the illumination of 200 commercial LEDs. Nanosecond laser processing enhances TENG performance through two key mechanisms: first, by generating an oxidized graphite layer on the PI surface, which facilitates charge storage and transfer; and second, by increasing the contact area between the PVDF triboelectric layer and the PI intermediate layer, improving charge transfer efficiency. The interplay between the laser-processed PI layer thickness, surface byproducts, PVDF friction layer thickness, and interfacial bonding significantly influences triboelectric charge generation, storage capacity, maximum output, lifespan, and output characteristics. Laser power primarily affects output by modulating the formation of surface byproducts on the PI layer, while scanning speed influences performance by altering the intermediate layer’s thickness. Previous studies have demonstrated that incorporating an intermediate layer with charge storage capabilities effectively enhances TENG output. This work advances that concept by leveraging ultraviolet nanosecond laser processing to simultaneously create microstructures and functional charge storage layers within the intermediate layer. This study provides a new perspective on the role of intermediate layers in TENG design and introduces a scalable method for enhancing both output performance and longevity. The insights gained from this study pave the way for future advancements in self-powered sensing systems, wearable electronics, and sustainable energy harvesting technologies.

## Supplementary Material



## Notes

Laser power and the processing morphology under a Super-Depth-of-Field Microscope, the outputs corresponding to the LMI-TENGs with different patterns; SEM images of the overlapping parts of the laser scanning path; the linear velocity of laser processing and the processing morphology under a superdepth-of-field microscope; SEM images of the junction between the intermediate layer and the triboelectric layer; LMI-TENG with gaps after spin-coating; stress simulation of the triboelectric layer and the intermediate layer of the LMI-TENG (PDF)
