# Supplementary material for: Enhancing Triboelectric Nanogenerator Performance via Ultraviolet Nanosecond Laser-Engineered Microstructured Intermediate Layer
Source: ACS Appl Mater Interfaces. 2025 Sep 18;17(39):54888–904. doi: 10.1021/acsami.5c13450 (PMC12492327; doi:10.1021/acsami.5c13450)
Supplement: Supplementary file 1 [file am5c13450_si_001.pdf]

Supporting Information

Enhancing Triboelectric Nanogenerator Performance via Ultraviolet Nanosecond Laser-Engineered Microstructured Intermediate Layer

*Zheng Zheng,[1] Lei Li,[1] * MingMing Liu,[1] Liyong Wang,[1] Ruiyong Yang,[1] Rui Li,[1] Langping Wang[3], Hongyu Zheng,[1] * Youbin Zheng[2] **

[1]School of Mechanical Engineering, Shandong University of Technology, Zibo, 255000, China.

[2]Department of Electrical Engineering and Electronics, University of Liverpool, Liverpool, L69 7GJ, UK

[3]State Key Laboratory of Precision Welding & Joining of Materials and Structures, Harbin Institute of Technology, Harbin, 150001, China.

*Corresponding Author: youbin.zheng@liverpool.ac.uk; zhenghongyu@sdut.edu.cn; lilei@sdut.edu.cn

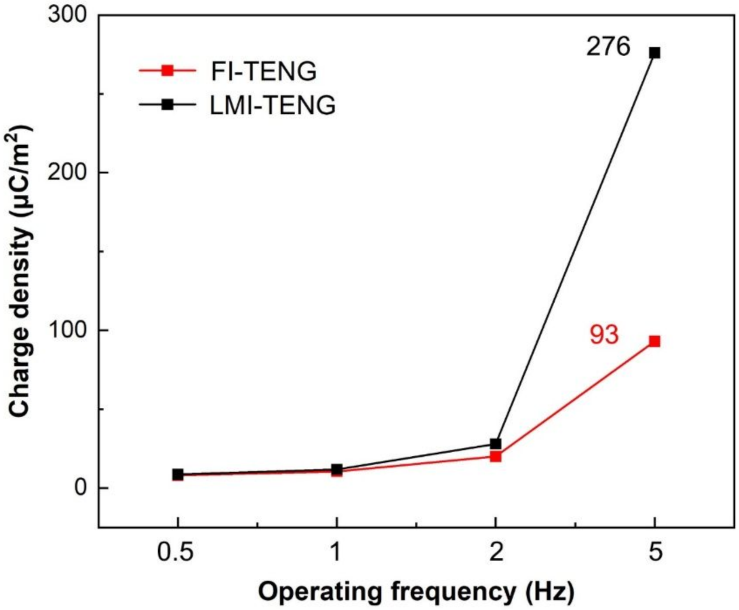

Figure S1. TENG operating frequency and dielectric layer surface charge density

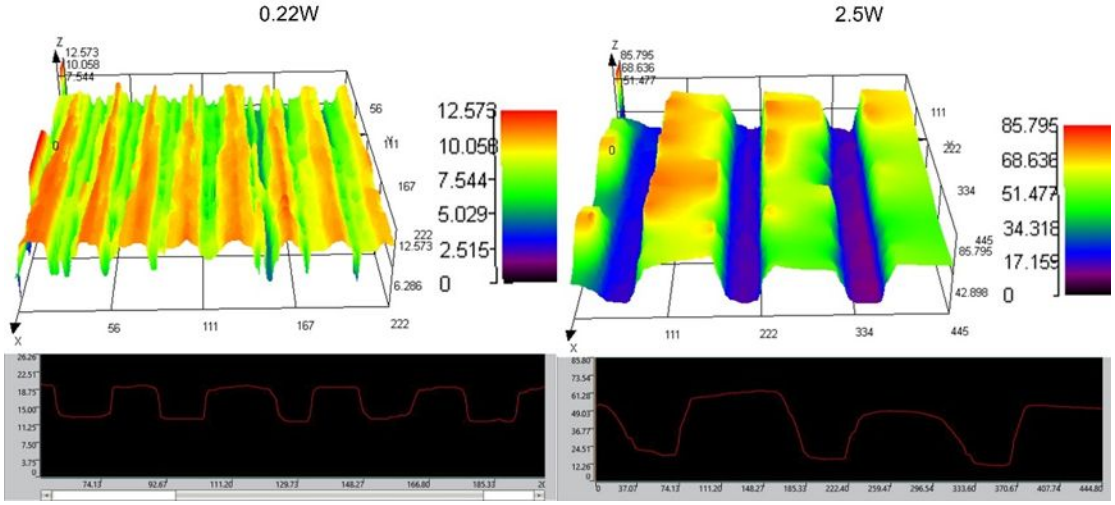

Figure S2. Laser power and the processing morphology under a Super-Depth-of-Field Microscope.

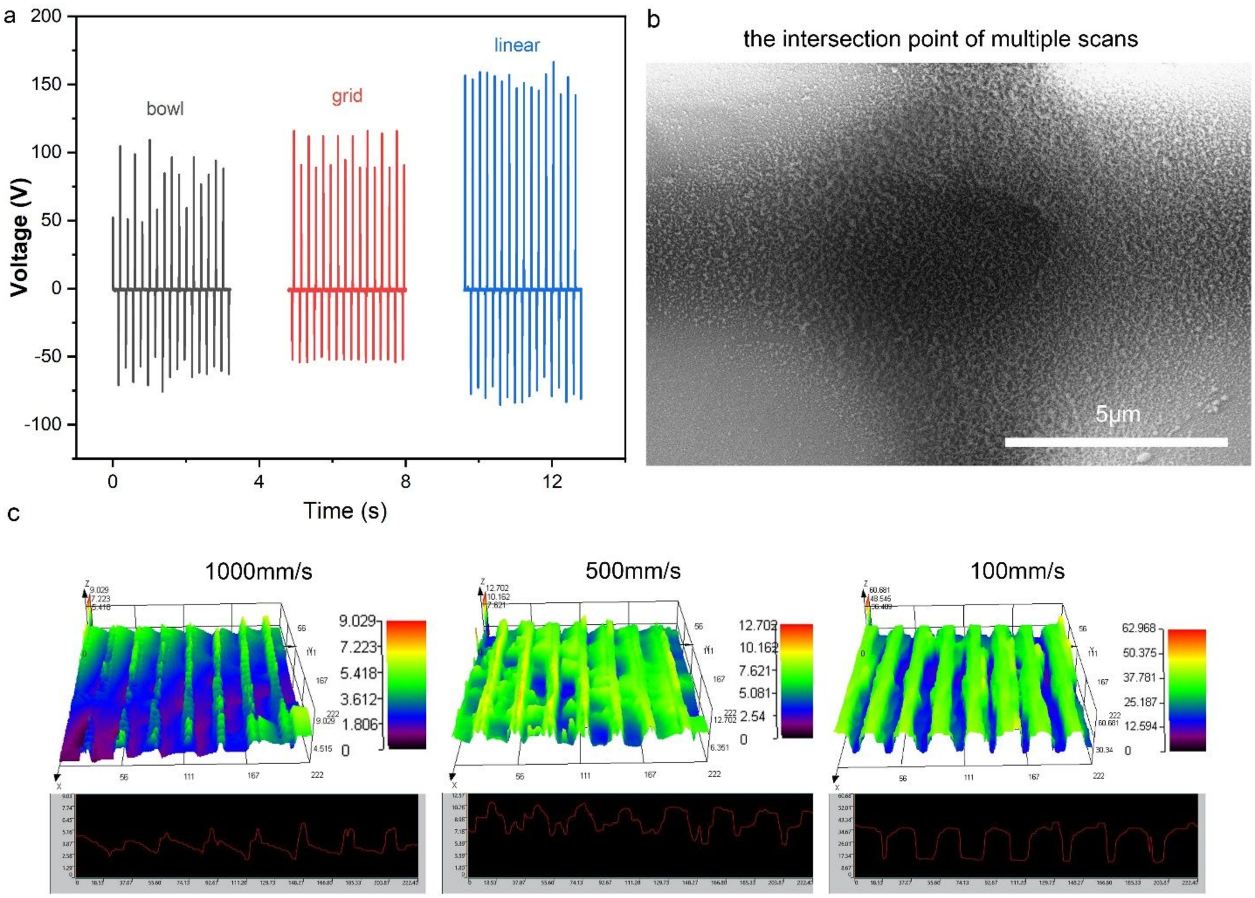

Figure S3. (a) The outputs corresponding to the LMI-TENGs with different patterns. (b) SEM image of the overlapping parts of the laser scanning path. (c) The linear velocity of laser processing and the processing morphology under a super-depth-of-field microscope.

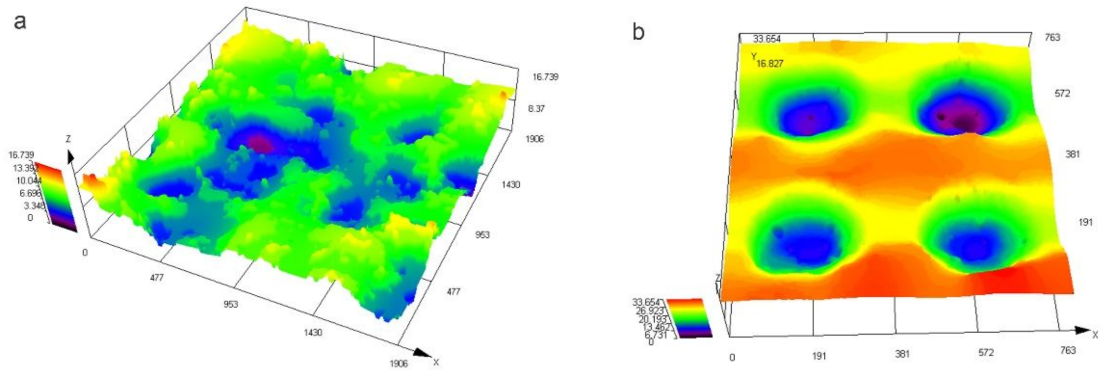

Figure S4. Super-depth-of-field views of structures with different size scales fabricated on 100 μm PI film. (a) PI patterned by single-pulse processing, (b) Structures with large depth and high quality fabricated by multi-pulse processing.

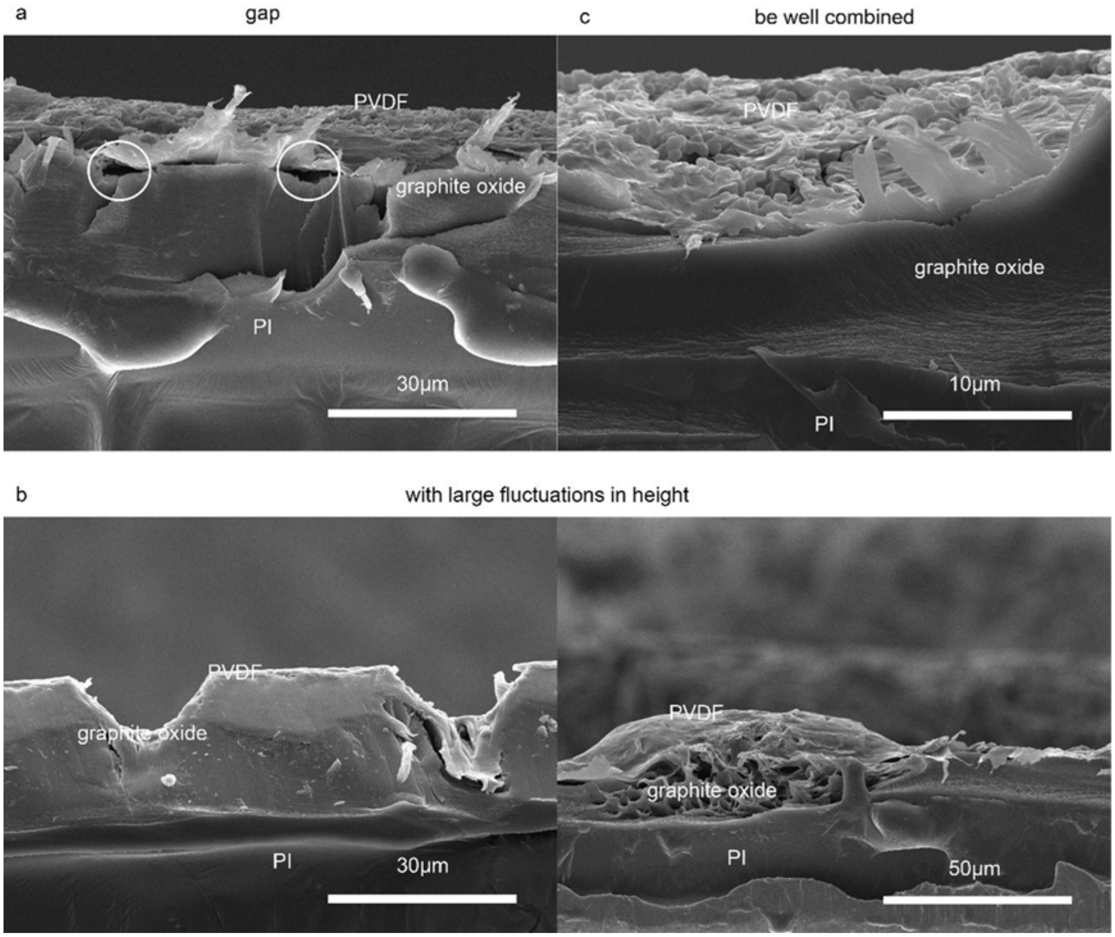

Figure S5. SEM images of the junction between the intermediate layer and the triboelectric layer. (a) Gap defects caused by the mismatch of the spin-coating speed. (b) Large fluctuations caused by high laser power (c) The spin-coating speed and the laser power are well matched.

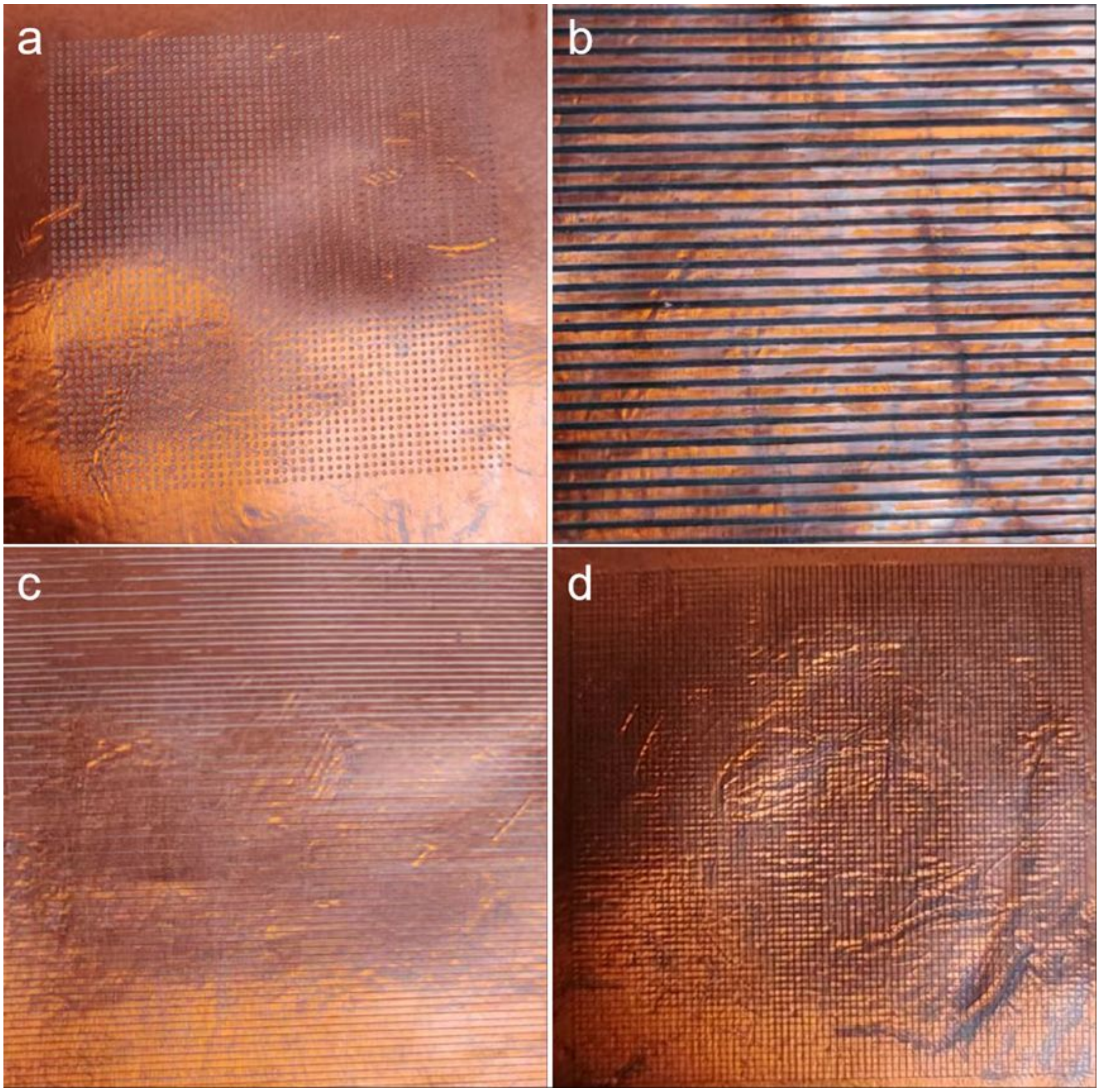

Figure S6. Typical LMI-TENG with gaps after spin-coating: (a) Bowl-shaped structure middle layer, (b) and (c) Linear structure middle layers, (d) Grid middle.

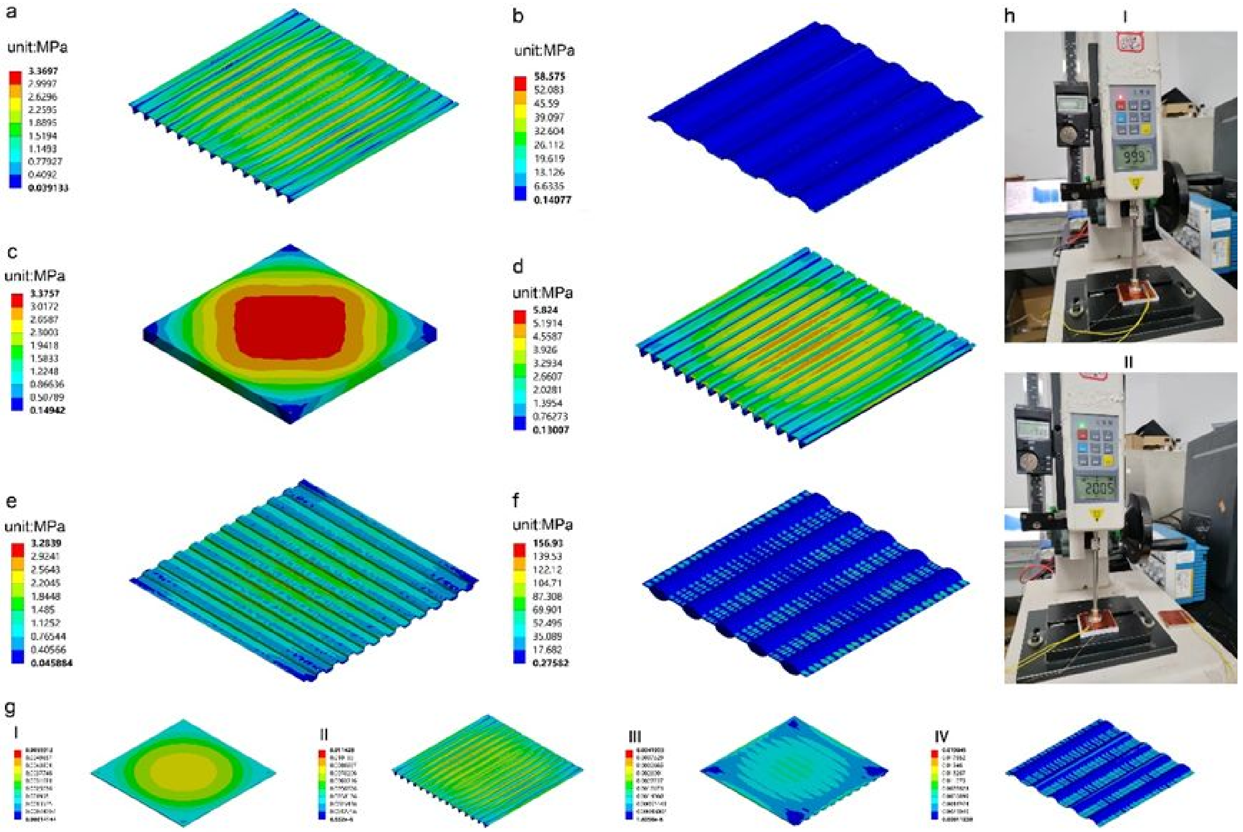

Figure S7. Stress simulation of the triboelectric layer and the intermediate layer of the LMI-TENG. (a) and (b) show the equivalent stress diagrams of the PVDF layer. (c-f) are the equivalent stress diagrams of the intermediate layer/graphite layer, (c) FI-TENG, (d) and (f) are LMI-TENGs, (e) is LMI-TENG with good bonding. (g) presents the equivalent strain, (I) FI-TENG, (II) and (III) are LMI-TENGs with poor bonding, while (IV) is LMI-TENG with good bonding. (h) shows the strain experiments under different pressures.

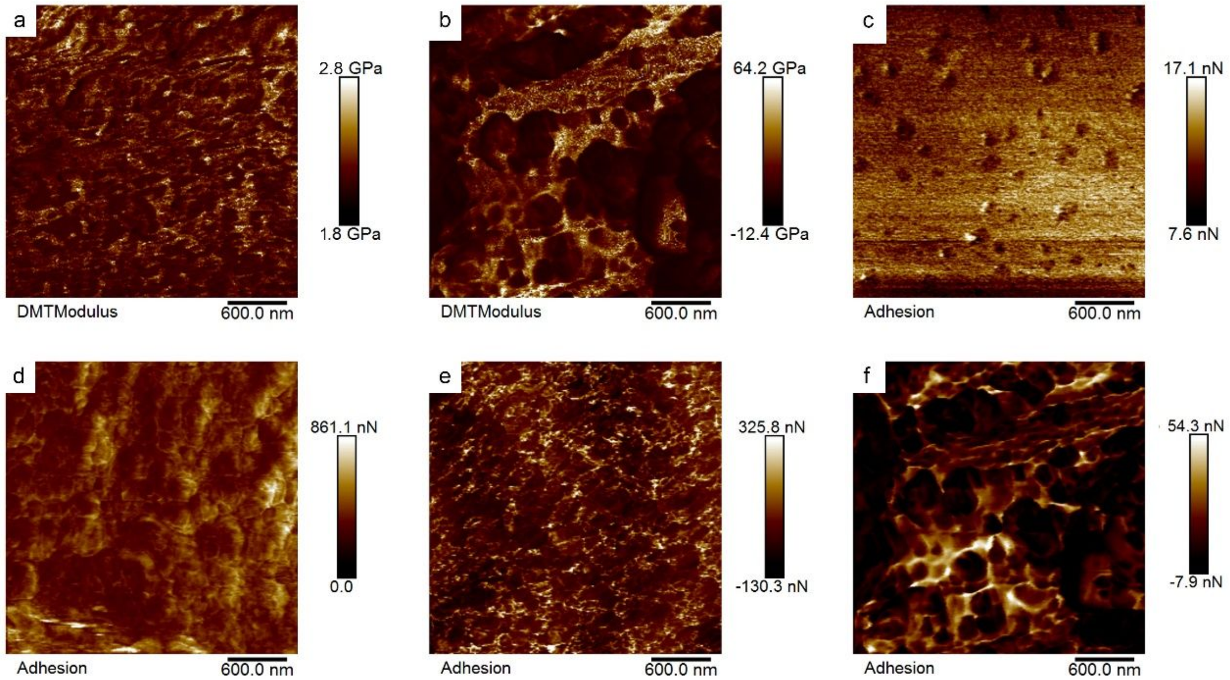

Figure S8. (a) is the 2D map of Young's modulus for the cross-section processed by 0.22 W laser; (b) is the 2D map of Young's modulus for the cross-section processed by 1.5 W laser; (c) is the surface adhesion of the PI; (d), (e), and (f) respectively represent the surface adhesion after laser processing at 0.22 W, 0.8 W, and 1.5 W.

Table S1. Effect of laser power on surface structure, composition, young's modulus of PI and TENG output performance (100 μm PI, 900 rpm spin-coating speed, 5 Hz frequency)

Laser power (W)	PI surface composition	Young's modulus	PI surface structure and TENG output (V)	cycling lifespan and maximum test pressure(N)
0.15W	Physical coalescence		Reduced output compared to FI-TENG	80N
0.22W	Carbonization and indirect formation of graphite oxide layer	4.8GPa	72V	430,000 (80N)
0.8W	In-situ formation of graphite oxide layer	11.4GPa	(linear) 154V (grid) 102V (bowl) 120V	80N
1.5W	In-situ formation of graphene oxide layer	179.3GPa	74V	260,000 (40N)
4.2W	In-situ formation of porous graphene		17V	20N
